# Genome-wide analysis reveals population structure and selection in Chinese indigenous sheep breeds

**DOI:** 10.1186/s12864-015-1384-9

**Published:** 2015-03-17

**Authors:** Caihong Wei, Huihua Wang, Gang Liu, Mingming Wu, Jiaxve Cao, Zhen Liu, Ruizao Liu, Fuping Zhao, Li Zhang, Jian Lu, Chousheng Liu, Lixin Du

**Affiliations:** Institute of Animal Sciences, Chinese Academy of Agricultural Sciences, National Center for Molecular Genetics and Breeding of Animal, Beijing, People’s Republic of China; National Animal Husbandry Service, National Center of Preservation and Utilization of Animal Genetic Resources, Beijing, People’s Republic of China

**Keywords:** Genome-wide analysis, China indigenous sheep, Population analysis, Selection signal analysis

## Abstract

**Background:**

Traditionally, Chinese indigenous sheep were classified geographically and morphologically into three groups: Mongolian, Kazakh and Tibetan. Herein, we aimed to evaluate the population structure and genome selection among 140 individuals from ten representative Chinese indigenous sheep breeds: Ujimqin, Hu, Tong, Large-Tailed Han and Lop breed (Mongolian group); Duolang and Kazakh (Kazakh group); and Diqing, Plateau-type Tibetan, and Valley-type Tibetan breed (Tibetan group).

**Results:**

We analyzed the population using principal component analysis (PCA), STRUCTURE and a Neighbor-Joining (NJ)-tree. In PCA plot, the Tibetan and Mongolian groups were clustered as expected; however, Duolang and Kazakh (Kazakh group) were segregated. STRUCTURE analyses suggested two subpopulations: one from North China (Kazakh and Mongolian groups) and the other from the Southwest (Tibetan group). In the NJ-tree, the Tibetan group formed an independent branch and the Kazakh and Mongolian groups were mixed. We then used the *d*_*i*_ statistic approach to reveal selection in Chinese indigenous sheep breeds. Among the 599 genome sequence windows analyzed, sixteen (2.7%) exhibited signatures of selection in four or more breeds. We detected three strong selection windows involving three functional genes: *RXFP2*, *PPP1CC* and *PDGFD. PDGFD*, one of the four subfamilies of *PDGF*, which promotes proliferation and inhibits differentiation of preadipocytes, was significantly selected in fat type breeds by the *Rsb* (across pairs of populations) approach. Two consecutive selection regions in Duolang sheep were obviously different to other breeds. One region was in OAR2 including three genes (*NPR2*, *SPAG8* and *HINT2*) the influence growth traits. The other region was in OAR 6 including four genes (*PKD2*, *SPP1*, *MEPE*, and *IBSP*) associated with a milk production quantitative trait locus. We also identified known candidate genes such as *BMPR1B*, *MSRB3*, and three genes (*KIT*, *MC1R*, and *FRY*) that influence lambing percentage, ear size and coat phenotypes, respectively.

**Conclusions:**

Based on the results presented here, we propose that Chinese native sheep can be divided into two genetic groups: the thin type (Tibetan group), and the fat type (Mongolian and Kazakh group). We also identified important genes that drive valuable phenotypes in Chinese indigenous sheep, especially *PDGFD*, which may influence fat deposition in fat type sheep.

**Electronic supplementary material:**

The online version of this article (doi:10.1186/s12864-015-1384-9) contains supplementary material, which is available to authorized users.

## Background

Sheep (*Ovis aries*), primarily raised for meat, wool, milk, and pelts, are an important part of the agricultural economy worldwide. It is the first grazing animal known to have been domesticated [1]. Archaeological evidence suggests that sheep were probably first domesticated in the Fertile Crescent, approximately 11,000 years ago [2]. China is one of the nine independent food producers worldwide [3], and has a long history of sheep husbandry. The earliest record of Chinese sheep remains can be traced back to approximately 5000–7000 years ago [4,5].

China has a great diversity of ecosystems and an abundance of sheep resources. Based on geographical distribution and morphological characteristics, there are 42 indigenous sheep breeds in China, which can be classified into three groups: Mongolian, Kazakh, and Tibetan [6]. The native domestic sheep breeds are highly adapted to local environmental conditions, and their most distinctive feature is the type of tail. Mongolian (fat-tailed) sheep are abundant in high latitudes; Kazakh (fat-rumped) sheep have the ability to deposit a large amount of fat in the body to meet nutritional demands during the winter and spring; The Tibetan (thin-tailed) sheep are generally present at low latitudes (southern area) where it is warmer and experiences less snow. In addition, artificial selection is also an important driving force for the formation of species diversity. Most of the Chinese domestic sheep are reared for meat, while some varieties are multipurpose. For instance, Duolang is an excellent mutton producer, Tibetan sheep are one of the major breeds for carpet wool in China, and Hu and Large-Tailed Han are prolific lambskin-type breeds. In a previous microsatellite analysis, Ma et al. [7] indicated that Chinese northern sheep could be divided into two broadly defined lineages, Tibetan origin and Mongolia origin, representing different geographical clusters. Zhong et al. [8] revealed three major clusters of Chinese indigenous sheep (Mongolian, Kazakh and Tibetan) and pointed out that Chinese indigenous sheep have a complicated genetic structure under the effects of different breeding histories, geographical distributions and ecological factors.

In the present study, we investigated 10 Chinese indigenous breeds that represent the main sheep types in China using the Illumina Ovine SNP50 Genotyping BeadChip. Our goal was to analyse the population structure and genome selection among Chinese native sheep breeds.

## Results

### Genetic variation and population genetic analysis

In this study, four metrics were used to estimate levels of within-breed genetic diversity (Additional file 1: Table S1). The polymorphism (*P*_n_), expected heterozygosity (*H*_*e*_), observed heterozygosity (*H*_*o*_), and inbreeding coefficients (F) among ten sheep populations were 0.9283–0.9675, 0.3278–0.3548, 0.3211–0.3526, and 0.0358–0.1234, respectively. Levels of polymorphism were generally high, more than 92% of loci displaying polymorphism within each population. The values of expected heterozygosity were close to observed heterozygosity in all populations, and the Duolang population had the lowest values for heterozygosity. Moreover, the *H*_*e*_ of Plateau-type Tibetan and Valley-type Tibetan presented were consistent with previous reports [9]. The lowest inbreeding coefficients were observed in Kazakh sheep (F = 0.0358). We then estimated the ranges of minor allele frequency (MAF) for all sheep breeds (Additional file 2: Figure S1). In all breeds, about 40% of single nucleotide polymorphisms (SNPs) are highly variable (MAF > 0.3) and 15% exhibit limited variability (MAF < 0.1). Finally, we investigated the extent of linkage disequilibrium (LD), as estimated by the average distance between SNPs that correspond to different linkage disequilibrium r^2^ (0.1–0.6) in each breed (Additional file 3: Table S2). The range of average distance was estimated as 236.26–155.11 kb and decreased with the increasing of r^2^ value. And the standard deviation was highest when r^2^ = 0.6.

**Figure 1:**
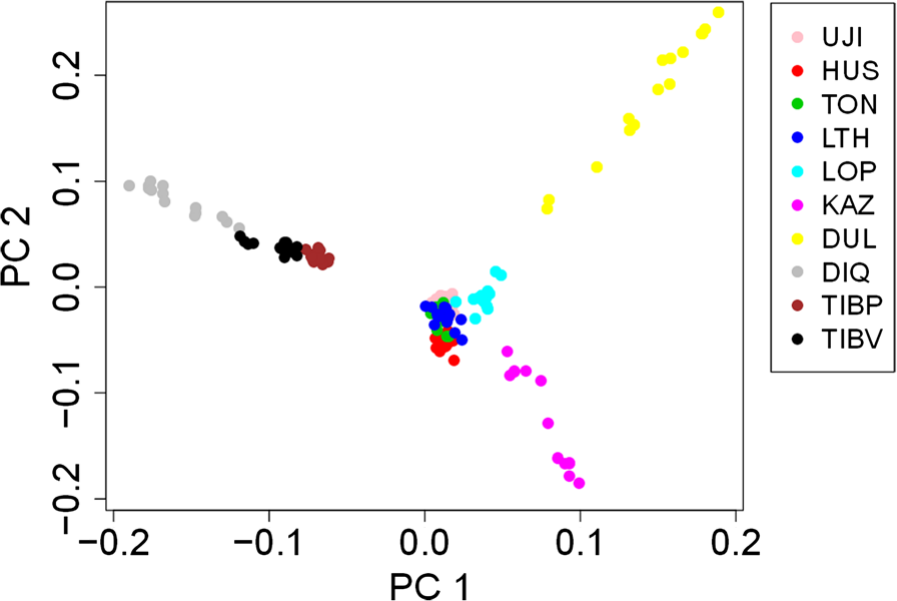
**Animals clustered on the basis of principal component (PC) analysis using individual genotypes.** Plot for the first (PC1) and second (PC2) component revealed the clustering of 140 animals from UJI, HUS, TON, LTH, LOP, KAZ, DUL, DIQ, TIBP, and TIBV breeds; UJI: Ujimqin sheep, HUS: Hu sheep, TON: Tong sheep, LTH: Large-tailed Han sheep, LOP: Lop sheep, KAZ: Kazakh sheep, DUL: Duolang sheep, DIQ: Diqing sheep, TIBP: Plateau-type Tibetan sheep, and TIBV: Valley-type Tibetan sheep.

To further examine the relationships among individuals, we performed principal components analysis (PCA) among all individuals (Figure 1). The analysis showed two principal components (PC1 and PC2), with variances of 3.2% and 2.6% (Additional file 4: Figure S2), respectively. According to PC1, Chinese sheep could be divided into two groups consistent with their fat deposition: thin-type sheep (PC1 < 0, Diqing, Plateau-type Tibetan, and Valley-type Tibetan) and fat-type sheep (PC1 > 0, Ujimqin, Hu, Tong, Large-Tailed Han, Lop, Duolang and Kazakh). They are further subdivided into two different sub-groups distinguished by differences in fat deposition. Second, combining the two principal components (PC1 and PC2) clustered the three Tibetan sheep (Diqing, Plateau-type Tibetan, and Valley-type Tibetan) together and the five Mongolian sheep (Ujimqin, Hu, Tong, Large-Tailed Han, and Lop) together; however, the two Kazakh sheep (Duolang and Kazakh) were clearly segregated (Figure 1). We could distinguish the three breeds in the Tibetan group, but it was more difficult in the Mongolian group, which was mixed together (Figure 1).

**Figure 2:**
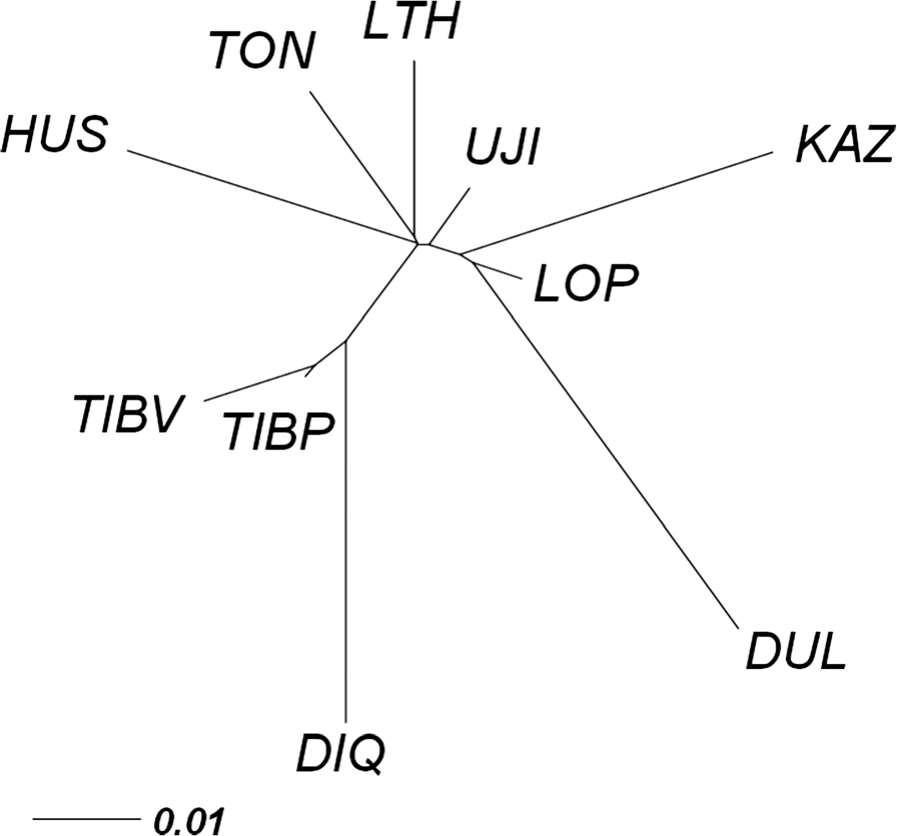
**Neighbor-Joining (NJ) phylogeny for 10 Chinese indigenous sheep breeds based on Pairwise** ***F***_***ST***_ **.** UJI: Ujimqin sheep, HUS: Hu sheep, TON: Tong sheep, LTH: Large-tailed Han sheep, LOP: Lop sheep, KAZ: Kazakh sheep, DUL: Duolang sheep, DIQ: Diqing sheep, TIBP: Plateau-type Tibetan sheep, and TIBV: Valley-type Tibetan sheep.

All pairwise *F*_*ST*_ values, which were rescaled as *F*_*ST*_ / (1- *F*_*ST*_), were calculated between the ten populations (Additional file 5: Table S3). The lowest level of differentiation was found between the Plateau-type Tibetan and Valley-type Tibetan populations (0.012), while the greatest divergence was observed between Diqing and Duolang (0.09). In the Mongolian sheep populations, *F*_*ST*_ between Lop and Ujimqin was the lowest (0.015). In comparison, the pairwise *F*_*ST*_ within the groups showed a closer relationship than between the groups. We calculated the mean pairwise *F*_*ST*_ (MPF) of each breed, which indicated that Ujimqin had the lowest MPF (0.033) and Duolang had the largest MPF (0.063). The NJ-tree showed clearly defined clusters (Figure 2). Diqing, Plateau-type Tibetan, and Valley-type Tibetan breeds were found in one main branch. This result was well supported by the traditional classification (Tibetan group) and their close distribution in the neighboring areas of Tibetan and Qinghai provinces. The other main lineage of the NJ tree included the Mongolian and Kazakh group. In addition, three breeds (Dulang, Lop, and Kazakh) from Xinxiang clustered in the same branch. We also constructed a neighbor-joining (NJ) tree among the 140 individuals (Additional file 6: Figure S3). The results were similar to the NJ-tree of the populations and clearly showed that there were no conflicts concerning the origins of individuals assigned to each breed.

**Figure 3:**
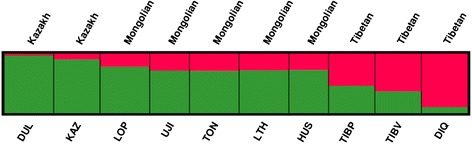
**Population structure of 140 sheep inferred by model-based clustering using STRUCTURE.** Results from *K* = 2 are shown.

To confirm our observation of the degree of divergence, the program STRUCTURE was applied to estimate the proportion of common ancestry among the 10 breeds. A model-based unsupervised hierarchical clustering of the individuals was analyzed by considering different *K* numbers (2–10) of predefined clusters based on 20,334 autosomal SNPs. The results of Bayesian clustering for *K* = 2 indicated that there was a clear transition from the Northwest and North China populations (green) to the Southwest and South China populations (red). We found that fat-type sheep were green dominated, wherein Duolang and Kazakh accounted for more than 90%. The thin-type sheep were red dominated, and Diqing accounted for 90% (Figure 3). This is also consistent with the PCA and NJ-tree. Furthermore, when the *K* value became large, some breeds were independent (Additional file 7: Figure S4). At *K* = 3, Duolang tended to be separated from the fat-type group. In the pairwise *F*_*ST*_ analysis, the mean pairwise *F*_*ST*_ of Duolang was the highest among the ten Chinese indigenous sheep breeds (Additional file 5: Table S3). Thus, Duolang might be a population subdivision within the Kazakh group. At *K* = 5, the Kazakh breed separated from the fat-type group. Soon after, Large-Tailed Han, Hu, and Tong separated one after another from the fat-type group when *K* = 8–10.

### Signatures of selection in the ovine genome

The *d*_*i*_ statistic is a new population-genomics strategy based on levels of population differentiation, which determines robustly whether selection acts on newly arisen or pre-existing variations [10]. We performed *d*_*i*_ statistic analyses to identify candidate regions targeted by selection in 10 Chinese indigenous sheep breeds. The *d*_*i*_ statistic was calculated for autosomal SNPs in 300-kb windows, with a minimum of three SNPs per window, and defining the populations by breed. The *d*_*i*_ statistic is a summation at each window of pairwise *F*_*ST*_ values for each breed combination, corrected by the value expected from genome-wide calculations; therefore, a large value of *d*_*i*_ statistic indicates greater divergence at that 300-kb window than that observed across the genome as a whole. In total, 46,618 SNPs were evaluated within 7738 windows, averaging 5.95 SNPs per window (SD = 1.6). We define candidate selection regions that fell into the upper 99^th^ percentile of the empirical distribution. The 78 windows within each breed were considered putative signatures of selection. In total, 599 of the windows met this criterion in one or more of the 10 breeds. These regions, considered putative signatures of selection in each breed, are listed in Additional file 8: Table S4. The maximum *d*_*i*_ statistic value per breed ranged from 16.32 in the Kazakh to 35.07 in the Valley-type Tibetan. Additional file 9: Figure S5 shows the genome-wide distribution of the *d*_*i*_ statistic.

**Figure 4:**
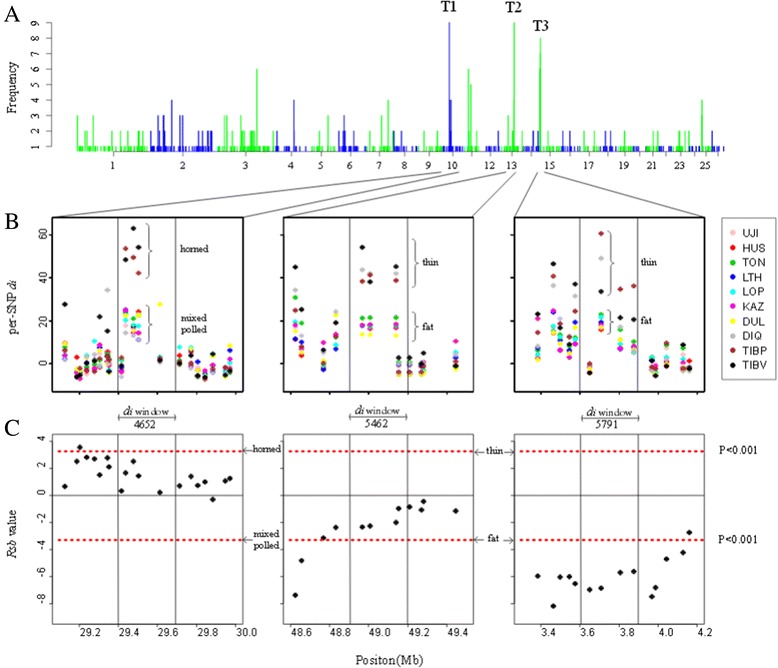
**Top 3 strongly selection regions in Chinese indigenous sheep. (A)** The number of overlapping signatures of selection in each 300-bp window. We defined an overlapping signature of selection for each window if the empirical P value was ≤ 0.01 in one breed. The black arrow indicates the chromosomal region shown in **B** and **C**; **(B)** The per-SNP *d*_*i*_ statistic of three regions with three consecutive windows, with T1, T2 and T3 widows in the middle. The braces indicate the group to which the breed belongs; **(C)** The *Rsb* value of each SNP in the three regions, which are consistent with **B**. The black arrow indicates the group name. The dashed red line denotes the significance threshold at P = 0.001.

To investigate how many selective events were unique or shared among breeds, we calculated the number of overlapping signatures of selection for each of the 155 significant 300-kb windows (Figure 4A). Sixteen of the 599 significant windows (2.7%) exhibited signatures of selection in four or more breeds. Sliding-window analyses of pairwise *F*_*ST*_ across the 300-kb interval suggested two or more independent selective events, reflected by two peaks of differentiation, with distinct patterns of allele frequency divergence among breeds (Figure 4A).

The first peak of differentiated region (T1, 4652 *d*_*i*_ statistic window) of the sheep genome showed evidence of selection in multiple breeds on OAR10 (Figure 4B). The differentiation observed from 29.4 to 29.7 Mb coincides with the *RXFP2* gene as a candidate gene for sheep horns [11]. In the 4652 window, we found that the 2^nd^ to 4^th^ SNP clearly divided 10 sheep breeds into two groups (Figure 4B), one is horned and the other is mixed and polled. The horned breeds (Plateau-type and Valley-type Tibetan) showed high levels of differentiation at *RXFP2* compared with the mixed and polled breeds (Figure 4B).

The second peak of differentiation region (T2, 5462 *d*_*i*_ statistic window, Figure 4B) in OAR13 from 48.9 to 49.2 Mb included the *PPP1CC* gene, also known as *PPP1G*, which is a subunit of the protein phosphatase 1. It is a glycogen-associated phosphatase responsible for dephosphorylation and subsequent inactivation of glycogen synthase and is universal in skeletal muscle [12]. *PPP1CC* is not required for insulin-stimulated glycogen synthesis in skeletal muscle, but appears to be a component of the response to contractile action [13,14]. Recent research indicates that *PPP1CC* is a positional candidate locus for skeletal muscle strength phenotypes [15]. In the 5462 *d*_*i*_ statistic window, three SNPs showed higher *d*_*i*_ statistic values in the thin-type breeds (Diqing, Plateau-type Tibetan, and Valley-type Tibetan) than in the fat-type breeds (Ujimqin, Hu, Tong, Large-tailed Han sheep, Lop, Kazakh, and Duolang) (Figure 4B).

We analyzed the remaining 14 significant regions, which showed selection between four and seven breeds, and found the T3 region (5791 *d*_*i*_ statistic window, Figure 4B) in OAR15, which was selected in seven breeds, and divided the 10 breeds into two groups, identical to the T2 region. This region, from 3.6 to 3.7 Mb, included only one functional gene: Platelet-derived growth factor D (*PDGFD*), which is one of the four subfamilies of *PDGF*, and is a potent stimulator of proliferation. PDGF was first identified [16] and purified [17] from human platelets, where it is sequestered in the alpha granules and released upon platelet activation. Studies have shown that the *PDGF* gene promotes proliferation and inhibits differentiation of preadipocytes [18,19]. Real-time quantitative PCR indicate that *PDGFD* is expressed at a higher level in adipose tissue e than in human normal tissues, except the thyroid [20].

To better understand the selection of these three regions, they were divided into two groups based on the *d*_*i*_ statistic value. *Rsb* (across pairs of populations) is a new approach to detect recent positive selection based on contrasting the extended haplotype homozygosity (*EHH*) profiles between populations [21]. We calculated the *Rsb* value based on the groups that were included in each window (Figure 4C). The *Rsb* results showed region T1 was selected in horned groups and region T2 was subject to selection in fat-type populations; however, neither reached statistical significance. Interestingly, we found some SNPs before T1 and T2 windows were under significantly selection in *Rsb* analysis. The density of Ovine 50 K SNP BeadChip is not high enough and may lead to low detection ability and deviation. By contrast, region T3 was significantly positively selected in the fat-type groups (P < 0.001) and the *PDGFD* gene promotes proliferation. Therefore, we hypothesized that this region may be related to the formation of fat-type sheep.

**Figure 5:**
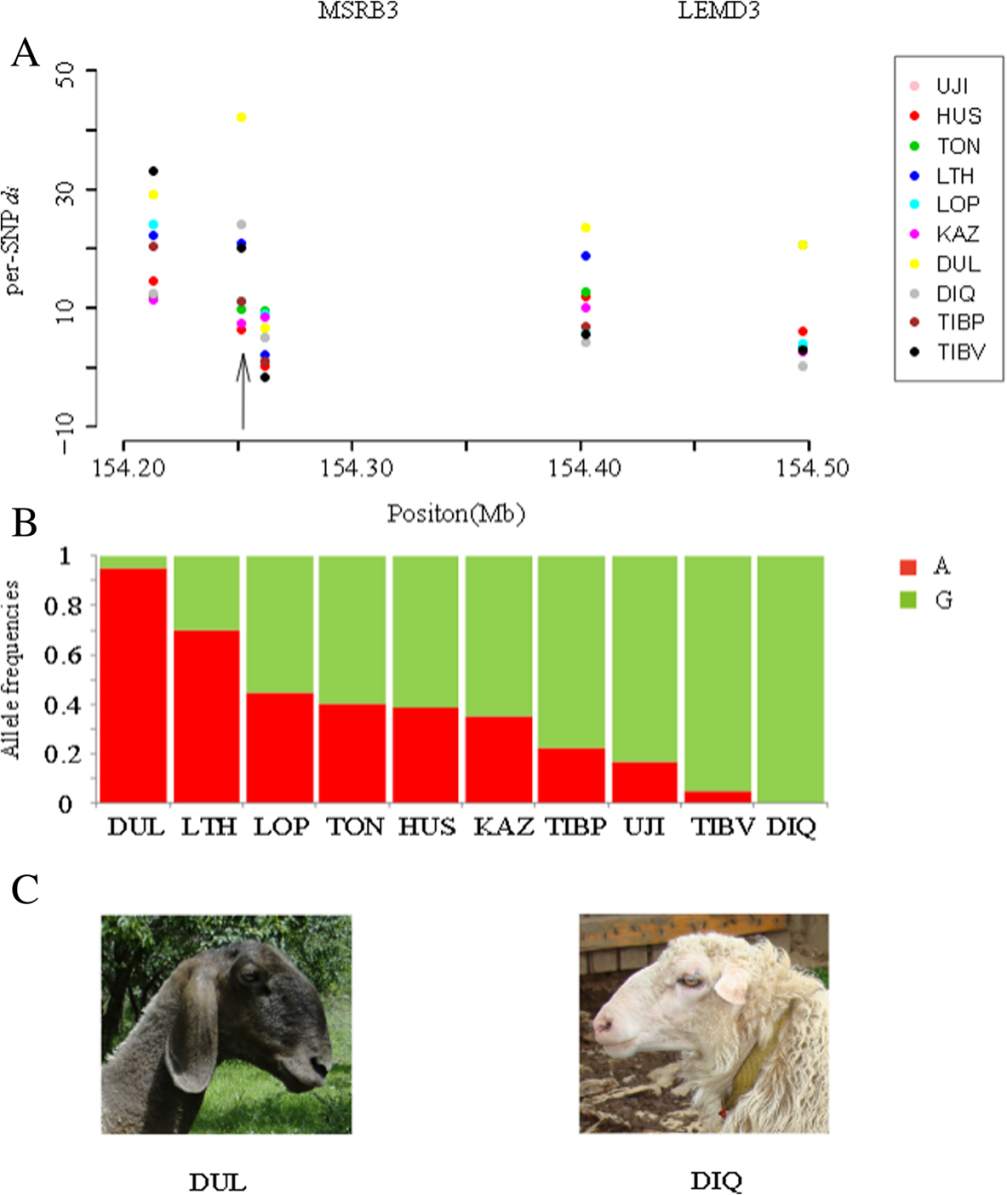
**A SNP associated with sheep’s ear morphology. (A)** The per-SNP *d*_*i*_ statistic in 154.2–154.5 Mb on OAR 3. The black arrow indicates a SNP that is associated with ear size; **(B)** The allele frequencies of 10 Chinese indigenous sheep breeds; red squares represents A and green squares represent G; **(C)** Duolang and Diqing: two extremes of the allele frequencies.

In addition, we also found an interesting peak of differentiation window in OAR3 of six selection breeds (Table 1), which overlaps with two genes, *MSRB3* and *LEMD3*. A SNP (OAR3_165050963.1) in this window, within MSRB3, differentiates Duolang strongly from the others (Figure 5A). *MSRB3* was identified as a candidate gene for ear morphology in dogs [22,23] and pigs [24,25] by genome-wide association studies (GWASs). At this locus, Duolang and Diqing show the two extremes of the allele frequencies (Figure 5B). From the pictures of each breed, we observed that the ears of the Duolang sheep are the largest of all the sheep breeds (Additional file 10: Table S5). Figure 5C shows photographs of the two extreme breeds. Therefore, we hypothesized that that this SNP is associated with ear size in sheep.

**Table 1:** **Annotation of the top six signatures of selection windows**

**Frequency**	**Breed**	**Region**	**Annotated Gene**
9	UJI, HUS, TON, LTH, LOP, KAZ , DUL, TIBP, TIBV	OAR 10: 29400000- 29700000	*EEF1A1,RXFP2*
	UJI, HUS, TON, LTH, LOP, KAZ , DIQ, TIBP, TIBV	OAR 13: 48900000- 49200000	*MUTED,PPP1CC*
7	UJI, TON, LOP, KAZ, DIQ, TIBP, TIBV	OAR 15: 3600000- 3900000	*PDGFD*
6	UJI, TON, LTH, LOP, DUL, TIBV	OAR 3: 154200000- 154500000	*MSRB3,LEMD3*
	UJI, LOP, DUL, DIQ, TIBP, TIBV	OAR 11: 18300000-18600000	*NF1,OMG, EVI2B*
	HUS, TON, LTH, DUL, DIQ, TIBP	OAR 15: 900000- 1200000	*AASDHPPT,ANKRD49,GPR83,LRRC28,MRE11A*

Breed abbreviations are described in Table 2.

**Table 2:** **Breeds included in the study and their distinguishing phenotypes**

**Breed**	**Location (province)**	**Group**	**Tail type**	**Horn type**	**Coat color**	**Breeding objective**	**Lambing percentage (%)**
Duolang (DUL)	Xinjiang	Kazakh	fat-rumped	polled	lamb brown, gray	meat-fat	250
Kazakh (KAZ)	Xinjiang	Kazakh	fat-rumped	males horned, ewes polled	brownish red	meat-fat	99
Lop (LOP )	Xinjiang	Mongolia	short fat-tailed	males horned, ewes polled	white	meat-fat	93
Ujimqin (UJI)	Inner Mongolia	Mongolia	short fat-tailed	males horned, ewes polled	white, black points	meat-fat	113
Hu(HUS)	Jiangsu	Mongolia	short fat-tailed	polled	white	meat-fat, lambskin	277
Tong (TON)	Shaanxi	Mongolia	long fat-tailed	polled	white	meat-fat	105
Large-tailed Han (LTH )	Shandong	Mongolia	long fat-tailed	males horned, ewes polled	white	meat-fat, lambskin	205
Plateau-type Tibetan (TIBP)	Qinghai	Tibet	thin-tailed	horned	white	meat, carpet wool	95
Valley-type Tibetan (TIBV)	Sichuan	Tibet	thin-tailed	horned	white , black points	meat, carpet wool	95
Diqing (DIQ)	Yunnan	Tibet	thin-tailed	males horned, ewes polled	white, black points	meat	95

All information comes from *ANIMAL GENETIC RESOURCES IN CHINA - SHEEP AND GOATS.*

### Cross traces in Duolang

**Figure 6:**
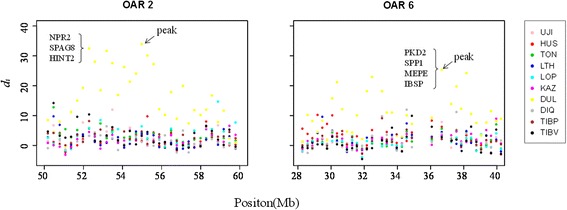
**Plot of** ***d***_***i***_ **windows of two regions on OAR2 (50–60 Mb) and OAR6 (28.2–42 Mb) of 10 Chinese indigenous sheep breeds.** A black arrow indicates the peak windows in each region. The braces include the candidate genes.

Duolang is a special breed that showed a large genetic distance from other Chinese indigenous breed in a previous population genetic analysis. Historically, a religious person brought Jill Wagner sheep from Afghanistan to Xinjiang in 1919, and then crossed them with native sheep breeds, eventually forming a new breed called Duolang [6]. The major characteristics are large body size, fast growth and a high reproductive rate [6]. In the present study, we found two consecutive selection regions on OAR2 and 6 (Figure 6) involving a total of 38 windows (11.4 Mb), which were almost half of the candidate selection regions of Duolang. These windows contained 68 candidate genes (Additional file 11: Table S6). In OAR2 (Figure 6), the highest *d*_*i*_ statistic window (*d*_*i*_ statisti*c* value = 33.69) did not include any genes, and the second highest *d*_*i*_ statistic window (value = 32.22) included nine genes (Additional file 11: Table S6). *NPR2*, which was found by both Kijas and Moradi [9,26], is involved in skeletal morphology and body size [27]. *SPAG8* and *HINT2* influence carcass weight and birth body weight, respectively [28]. In OAR6 (Figure 6), we found that the highest *d*_*i*_ statistic window (value =25.07) was for a region associated with a milk production QTL in cattle [29,30], which included four genes (*PKD2*, *SPP1* (also called *OPN*), *MEPE* and *IBSP*). In particular, *SPP1* is a candidate gene for the litter size in pig [31,32].

### Other important candidate genes selected in less than three breeds

Such universal differentiation at a single locus is consistent with the action of a gene that generally sorts individuals into phenotypic classes and breed groups. In this study, we also found some common selection genes that influence reproduction and coat color. *BMPR1B*, located on OAR6 (29.1–29.7 Mb), showed strong evidence of selection in highly prolific breeds Hu and Large-tail Han. Although *BMPR1B* is associated with high prolificacy in some Chinese breeds or strains of sheep, other researchers believe that *BMPR1B* is not the only gene responsible for the fecundity of Chinese sheep [33]. We also identified that *KIT*, *MC1R*, and *FRY* influence coat color. A region on OAR3 (44.6–45.6 Mb) that includes the *KIT* gene is associated with melanocyte development and accounts for pigmentation phenotypes in pigs and horses [34,35], which also shows a strong signature of selection in the Duolang and Kazakh breeds, both of which have dark coat colors (Additional file 10: Table S5). *MC1R* was selected in the Ujimqin population. A mutation in *MC1R* causes black spotting in pigs [36]. *FRY*, which is a key candidate gene involved in the piebald phenotype in merino sheep [37], is located on Chromosome 10 and was selected in Valley-type Tibetan sheep, whose coat has black spots (Additional file 10: Table S5). Interestingly, *FRY* was also considered as a candidate gene affecting wool quality between Rambouillet and Suffolk sheep [38]. Furthermore, *WNT6* and *WNT10A*, which were only selected in the rumped tail breeds Kazakh and Duolang, inhibit adipogenesis via a β-catenin-dependent mechanism [39,40].

## Discussion

In the present study, population genetic analysis was performed on 50 K SNP genotypes of 140 animals for 10 Chinese indigenous sheep breeds. We analyzed the population using PCA, STRUCTURE, and NJ-tree. The results indicated that Chinese indigenous sheep populations could be subdivided into two genetic clusters: the Tibetan group and the Mongolian and Kazakh group.

Overall, the partitioning of genetic diversities of the breeds is consistent with their geographic distributions. The Mongolian group is the most widely distributed breed in China, mainly in Inner Mongolia, the Central Plains, and eastern coastal areas. This is attributed to good adaptability and performance of the Mongolian sheep and Genghis Khan’s expedition in the Yuan dynasty [41]. The most abundant species diversity is in Xinjiang province, which is mainly attributed to its location. It is the only way to the trading venues in the “Silk Road” [42], and its position in Central Asia and the Central Plains, and the geographic isolation of its southern and northern parts is one reason for its rich diversity. The Tibetan group living in the southwest region, which is a mountainous region, also has abundant genetic diversity.

According to pairwise *F*_*ST*_, the relationships between Lop and Ujimqin (pairwise *F*_*ST*_ =0.015) and between Plateau-type Tibetan and Valley-type Tibetan (pairwise *F*_*ST*_ =0.012) were closer than others. These two combinations were observed to be mixed together in the PCA plot (Figure 1), and had a similar composition in STRUCTURE from *K* = 2–10 (Additional file 2: Figure S1). The greatest divergence was observed between Diqing and Duolang (0.09), which come from different groups: Diqing belongs to the Tibetan group and Duolang belongs to the Kazakh group. A previous study indicated that the Mongolian group was the origin of Tong sheep [43]. However, according to our study, Ujimqin is the oldest breed in the Mongolian population compared with Tong sheep, because Ujimqin not only has the smallest MPF and the highest polymorphism in the Mongolian group, but also it lives closer to Mongolia, the capital of the Yuan dynasty [41], geographically.

Population genetic analysis was able to distinguish physiological differences and geographical origins. In this study, Duolang and Kazakh come from Xinjiang province; however, their genetic difference was in the middle of the range of differentiation (pairwise *F*_*ST*_ = 0.069), and similar results were seen from PCA and STRUCTURE (Figures 1 and 2). One reason is that the Tianshan Mountains divide Xinjiang into southern and northern regions: Duolang comes from the southern region and Kazakh comes from the northern region. This results in the geographical isolation between Duolang and Kazakh. There is another explanation, according to record, Duolang was mixed with Afghanistan sheep [6,44], which may be the cause of this phenomenon. Interestingly, we found Lop, Duolang, and Kazakh clustered in a branch of the NJ-tree. However, Lop belongs to the Mongolian group, according to the PCA figures it is closer to Mongolian sheep breeds than Duolang and Kazakh. In STRUCTURE result when *K* = 4–7, Lop breed appears to be some introgressed of Kazakh group, but this needs more intensive research to explain.

These data provided strong evidence of the genetic structure in these 10 sheep breeds. In this study, which involved just a quarter of Chinese local breeds, there were very complex genetic relationships. For more accurate understanding of the relationships between them, more breeds should be analyzed.

### Selection in Chinese sheep breeds

Most studies of artificial selection in sheep have focused on single-gene analyses arising from phenotype-driven studies. Recently, Kijas et al. [9] analyzed 74 sheep breeds worldwide, one of which was a Chinese native breed, Tibetan sheep. The study identified the strongest selected candidate gene, *RXFP2*, in response to breeding for the absence of horns [9]. We also identified this gene in the peak of the differentiated region in the Chinese population. Most Chinese indigenous sheep are dual-purpose, low breeding breeds. Only a few specialized breeds are used for lambskin and lamb fur. Hu, Large-tailed Han and Tong are lambskin breed. Hu and Large-tailed Han are amongst the most prolific breeds in the world. *BMPR1B*, a notable candidate gene, was selected in both breeds. Other candidate genes focused on profile characteristics, such as *KIT*, *MC1R*, and *FRY*, which influence variation in dark coat color and the piebald phenotype.

The fat-tail is an important component in sheep. Currently, approximately 25% of the world sheep population comprises fat-tail breeds, which are found in a wide range of countries in Asia, the Middle East and North Africa [45]. In China, fat-tail breeds comprise 80% of the population. In terms of tail type, Chinese breeds are divided into four groups: short fat-tailed, long fat-tailed, fat-rumped, and thin-tailed (Table 2). The first three types of tail shape are not the same, but still belong to fat-type sheep. This trait is now commercially less important because of improved forage availability and decreased price of the product. Therefore, a decrease in fat-tail size is often desirable for Chinese producers. To date, several investigations into the inheritance of fat-tails have been undertaken. Moradi et al. [26] confirmed three regions located on Chromosomes 5, 7, and X that affect thin and fat tail breeds. Bakhtiarizadeh et al. [46] suggested that the expression of *FABP4* in the fat-tail is an important index of fat deposition. The haplotype in *CAST* can distinguish between fat-tailed and thin-tailed sheep breeds [47]. Recently, Wang et al. [48] identified 646 genes that were differentially expressed between fat-tailed and thin-tailed groups using RNA-seq, and identified *NELL1* and *FMO3* as genes relevant to fat metabolism in adipose tissues. Despite these studies, there is no compelling reason for the impact of a fat-tail on the formation mechanism. It is worth mentioning that the results of Moradi’s [26] and our are inconsistent, even if both of us used similar type of population and the Ovine 50 K SNP Bead chip. There include two reasons: Firstly, Chromosome X was not analysis in our study; Secondly, the version of sheep reference genome assembly are different, they used ver.1.0 from CSIRO [49] and we used the latest sheep genome release Ovis_aries_v3.1 (http://www.livestockgenomics.csiro.au/sheep/oar3.1.php). In this study, we found that two windows comprising the *PPP1CC* and *PDGFD* genes are associated with significant differences between fat-tail and thin-tail groups. Evidence shows that the first domesticated sheep were thin-tailed and that the fat-tail developed later [45]. As expected, *PDGFD* was strongly positively selected in fat-tail breeds by the *Rsb* approach. Moreover, the PDGF family promotes proliferation and inhibits differentiation of preadipocytes [18,19]. In addition, *PDGFD* is highly expressed in adipose tissue [20]. Until now, research on PDGFD has concentrated on its association with diseases, especially cancers in humans. Thus, we hypothesized that *PDGFD* plays an important role in sheep adipose tissue and is a candidate gene that may lead to the formation of the fat-tail.

## Conclusions

In this study, PCA, STRUCTURE and NJ-tree analysis both revealed that Chinese sheep populations could be subdivided into two genetic clusters. One is the Tibetan group (thin-tail) and the other is the Mongolian and Kazakh group (fat-tail). We suggest that Chinese indigenous sheep have descended from two ancestors, from Northwest and Southwest China, respectively.

We used the *d*_*i*_ to reveal selection in the sheep populations. We found known candidate genes such as *RXFP2*, *BMPR1B*, *MSRB3*, and *KIT*, *MC1R*, and *FRY*, which influence horn, lambing percentage, ear size, and coat phenotypes, respectively. We also detected two strong selection windows that split China sheep into fat-type (Mongolia and Kazakh group) and thin-type (Tibetan group). The *Rsb* approach identified a positively selected window that included a candidate gene, *PDGFD*, for formation of the fat-tail. Further research on the association of this gene with fat deposition in sheep will be performed.

## Methods

### Ethics statement

Blood sampling was approved by the Biological Studies Animal Care and Use Committee, Peoples Republic of China. The feeding was in line with the Instructive Notions with Respect to Caring for Laboratory Animals that was published in 2006 by the Science and Technology Department of China (Approval No. S20072911).

### DNA samples and SNP genotyping

For Chinese sheep breeds, blood samples were collected using traditional method from 12 Ujimqin sheep (UJI), 12 Hu sheep (HUS), 15 Tong sheep (TON), 15 Large-tailed Han sheep (LTH), 15 Lop sheep (LOP), 14 Kazakh sheep (KAZ), 15 Duolang sheep (DUL), 14 Diqing sheep (DIQ), 14 Plateau-type Tibetan sheep (TIBP), and 14 Valley-type Tibetan sheep (TIBV). These 10 Chinese indigenous breeds are mainly distributed in 8 provincial administrative regions (Inner Mongolia, Sichuan, Jiangsu, Shandong, Tibet, Yunnan, Xinjiang, and Qinghai), which represents the main sheep husbandry systems in China (Table 2). These animals had recently utilized the registration and recording system of NCPUGRDA (National Center for Preservation and Utilization of Genetic Resources of Domestic Animals, National Animal Husbandry service, Beijing, China). In general, 140 individuals were genotyped on the Illumina Ovine SNP 50 K Bead Chip assay at Capital Bio Corporation (Beijing, China).

### Quality control and genetic diversity analyses

SNPs that cannot pass the following three criteria were excluded: (1) SNPs with minor allele frequency (MAF) >0.01; (2) maximum per-SNP missing rate <0.05; (3) Hardy–Weinberg Equilibrium *P-*value >0.000001. After quality control, there were 140 individuals and 47,801 SNPs in the genetic diversity analysis dataset. The proportion of polymorphic SNP (***P***_**n**_) gives the fraction of total SNP that displayed both alleles within each population. Expected heterozygosity (*H*_*e*_), observed heterozygosity (*H*_*o*_), and inbreeding coefficient (F) were estimated by PLINK [50]. We also computed the r^2^ value between each marker pair within each breed separately using PLINK [50].

### Population analyses

Before analysis, we excluded SNPs on chromosome X, following which 46,618 SNPs were pruned using the indep-pairwise option, with a window size of 25 SNPs, a step of 5 SNPs, and r^2^ threshold of 0.05, resulting in 20,334 independent SNP markers. Principal component analysis (PCA) was conducted using using snpStats in R (http://cran.r-project.org). Population structure was evaluated using STRUCTURE 2.3.4 software [51]. All 140 animals were analyzed in triplicate for *K* = 2–10. All analyses were performed with a burn-in length of 20,000 followed by 30,000 MCMC replications for each *K*-value. To generate data files used in the CLUMPP 1.1.2 software [52].The solutions for all *K* were visualized using DISTRUCT 1.1 software [53]. Matrix pairwise *F*_*ST*_ value was estimated for all loci between populations using the Genepop 4.2.2 software [54], then rescaling *F*_*ST*_ as *F*_*ST*_ /(1- *F*_*ST*_) and the neighbor-joining tree for populations were construction with R package *ape* base on matrix pairwise rescaling *F*_*ST*_ values [55]. We also construct the neighbor-joining tree for individuals using SplitsTree software [56].

### Statistic analyses

Pairwise *F*_*ST*_ values per-SNP between breeds were calculated by Genepop 4.2.2 software [54]. Breed-specific population differentiation within 300 kb windows across the 26 autosomes was calculated using the statistic introduced by *d*_*i*_*statistic* [10]. Only windows with a minimum of 3 SNPs were considered. For each breed, the window of significance was determined as those with *d*_*i*_*statistic* values falling into the 99th percentile of the empirical distribution.

Using haplotype information, we computed *Rsb* and p_*Rsb*_ by R package *rehh* [57]. Haplotypes were estimated with *fastphase 1.4* [58]. We used population label information to estimate phased haplotype background and the following options for each chromosome: −Ku40 -Kl10 -Ki10. Per-SNP *Rsb* scores were transformed into p_*Rsb*_ = − log[Φ(*Rsb*)]. As above, assuming *Rsb* are normally distributed (under neutrality), *P*_*Rsb*_ might be interpreted as log_10_(1/P), where P is the one-sided P-value associated to the neutral hypothesis. SNP was considered significant if it exceeds the genome-wide significance threshold for *Rsb* (*P*_*Rsb*_ >3, *P*<0.001).

### Availability of supporting data

Supporting information is available in the additional files and further supporting data is available from the authors on request.

## Additional files

Additional file 1: Table S1. Genetic Diversity in 10 Chinese indigenous sheep population. Additional file 2: Figure S1. Minor allele frequencies (MAFs) of 10 Chinese indigenous sheep breeds. Additional file 3: Table S2. The average distance between SNPs of different r^2^ values. Additional file 4: Figure S2. Scree Plot of proportion of variance. Additional file 5: Table S3. Pairwise *F*_*ST*_ among 10 Chinese indigenous breeds. Additional file 6: Figure S3. Neighbor-Joining (NJ) phylogeny for 140 sheep. Additional file 7: Figure S4. (A) Population structure of 140 sheep inferred by admixture model-based clustering using STRUCTURE. Results from *K* = 2–10 are shown; (B) Posterior probability of the data given over 4 runs for each *K*; (C) Mean L(*K*) (±SD) over 4 runs for each *K* value. Additional file 8: Table S4. Details of the 599 candidate selection regions. Additional file 9: Figure S5. Genomic distribution of the population structure in 10 Chinese indigenous sheep breeds. The distribution of the *d*_*i*_ statistic for each 300-kp interval across all autosomes is shown for each breed. Alternating blue and green indicate values of the *d*_*i*_ statistic from adjacent chromosomes. The dashed red line denotes the 99th percentile for each breed. Additional file 10: Table S5. Images of 10 Chinese indigenous sheep breeds. Additional file 11: Table S6. Annotation of consecutive selection regions on OAR2 and OAR6 of Duolang sheep.
