# Supplementary material for: Genome-wide analysis reveals population structure and selection in Chinese indigenous sheep breeds
Source: BMC Genomics. 2015 Mar 17;16(1):194. doi: 10.1186/s12864-015-1384-9 (PMC4404018; doi:10.1186/s12864-015-1384-9)
Supplement: Additional file 1: Table S1. — Genetic Diversity in 10 Chinese indigenous sheep population. [file 12864_2015_1384_MOESM1_ESM.docx]

**Table S1 Genetic Diversity in 10 Chinese indigenous sheep population**

| *Breed* | ***n*** | ***P*_n_** | *H_e_* | *H_o_* | F |
| --- | --- | --- | --- | --- | --- |
| UJI | 12 | 0.9448 | 0.3489 | 0.3510 | 0.0395 |
| HUS | 12 | 0.9349 | 0.3367 | 0.3395 | 0.1087 |
| TON | 15 | 0.9535 | 0.3548 | 0.3360 | 0.0889 |
| LTH | 15 | 0.9499 | 0.3477 | 0.3319 | 0.1123 |
| LOP | 15 | 0.9675 | 0.3474 | 0.3260 | 0.0842 |
| KAZ | 14 | 0.9490 | 0.3429 | 0.3526 | 0.0358 |
| DUL | 15 | 0.9346 | 0.3278 | 0.3211 | 0.1234 |
| DIQ | 14 | 0.9283 | 0.3300 | 0.3253 | 0.1102 |
| TIBP | 14 | 0.9474 | 0.3417 | 0.3271 | 0.1069 |
| TIBV | 14 | 0.9350 | 0.3341 | 0.3365 | 0.0800 |

Note: genetic diversity indices measured within breed. n gives the number of individuals used to calculate the proportion of SNP displaying polymorphism (*P_n_*); expected heterozygosity (*H_e_*); observed heterozygosity (*H_o_*); the inbreeding coefficient (F). The abbreviations for the 10 breeds are shown in Table 1.
