# Supplementary figures and images for: Genome-wide analysis reveals population structure and selection in Chinese indigenous sheep breeds
Source: BMC Genomics. 2015 Mar 17;16(1):194. doi: 10.1186/s12864-015-1384-9 (PMC4404018; doi:10.1186/s12864-015-1384-9)

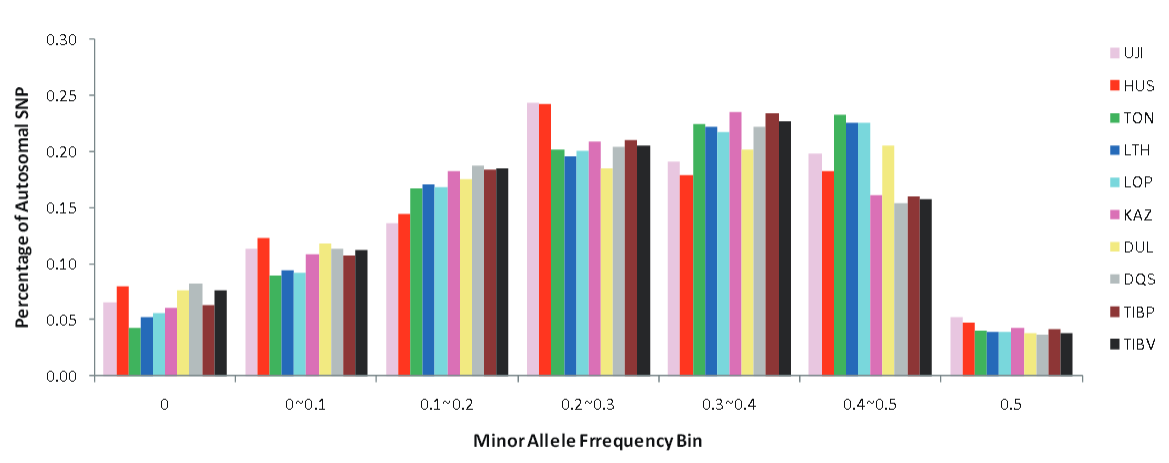

Supplement: Additional file 2: Figure S1. — Minor allele frequencies (MAFs) of 10 Chinese indigenous sheep breeds. [file 12864_2015_1384_MOESM2_ESM.png]

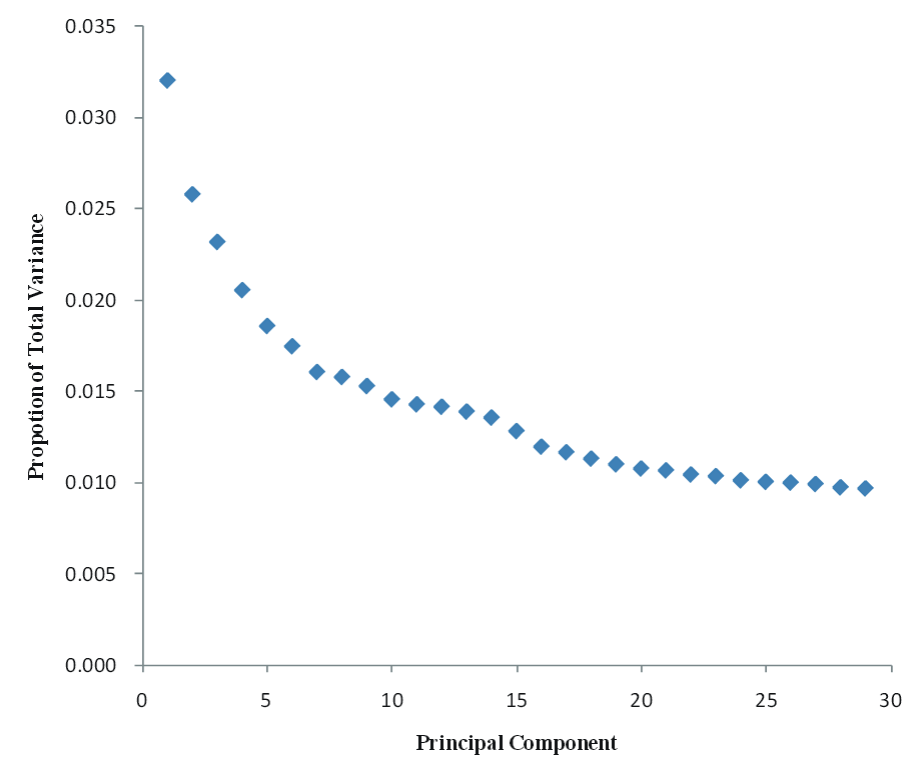

Supplement: Additional file 4: Figure S2. — Scree Plot of proportion of variance. [file 12864_2015_1384_MOESM4_ESM.png]

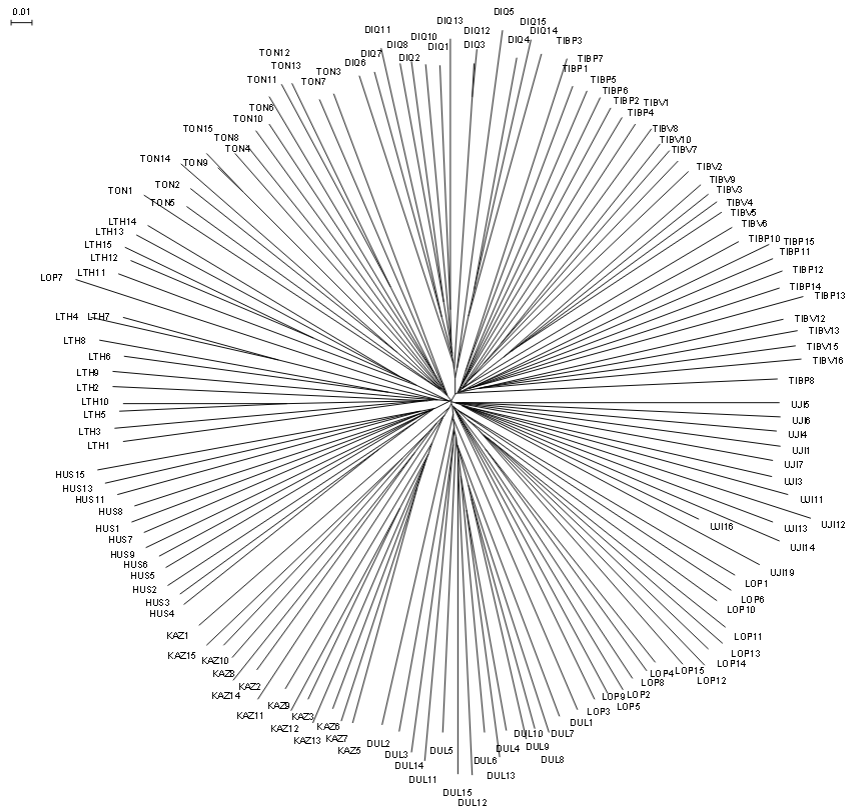

Supplement: Additional file 6: Figure S3. — Neighbor-Joining (NJ) phylogeny for 140 sheep. [file 12864_2015_1384_MOESM6_ESM.png]

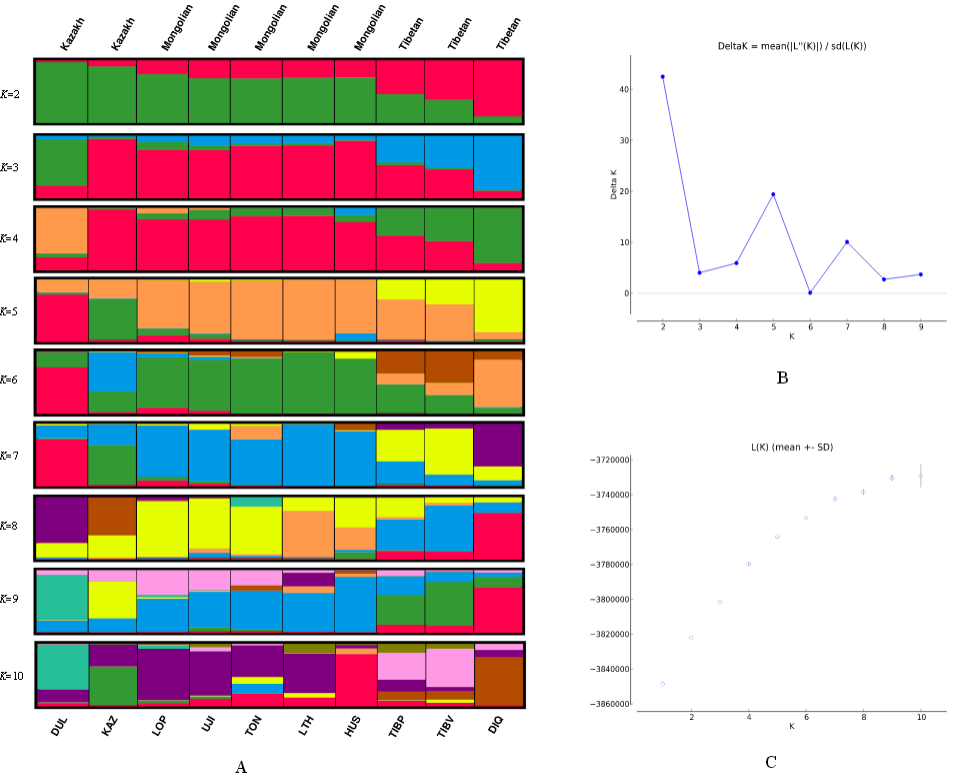

Supplement: Additional file 7: Figure S4. — (A) Population structure of 140 sheep inferred by admixture model-based clustering using STRUCTURE. Results from K = 2–10 are shown; (B) Posterior probability of the data given over 4 runs for each K; (C) Mean L(K) (±SD) over 4 runs for each K value. [file 12864_2015_1384_MOESM7_ESM.png]

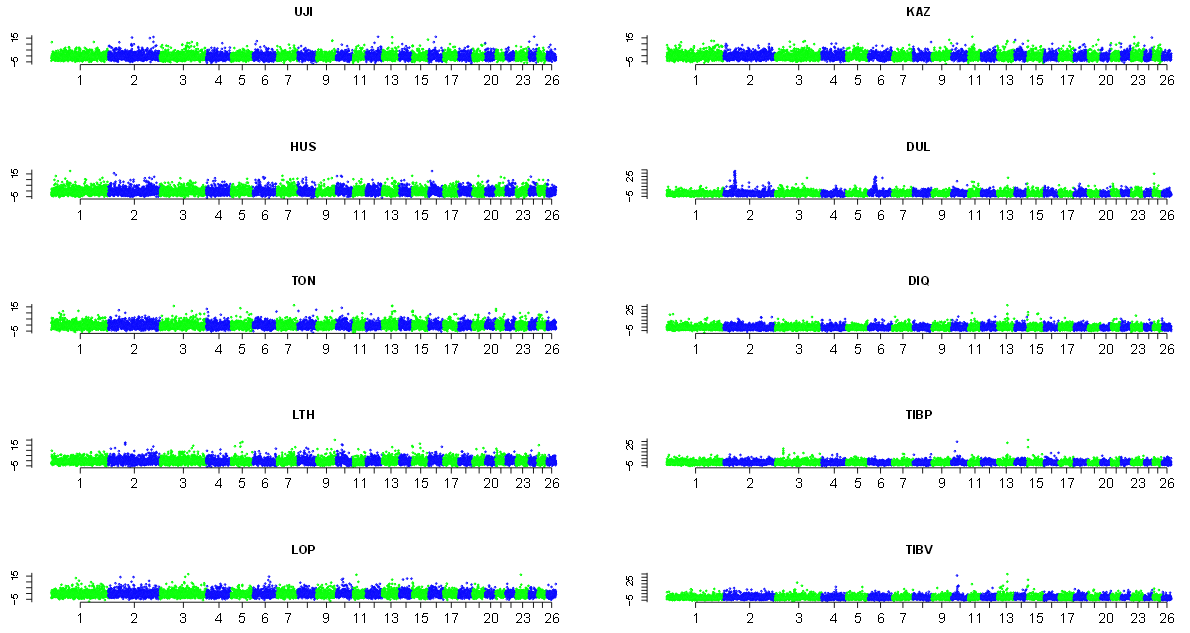

Supplement: Additional file 9: Figure S5. — Genomic distribution of the population structure in 10 Chinese indigenous sheep breeds. The distribution of the d i statistic for each 300-kp interval across all autosomes is shown for each breed. Alternating blue and green indicate values of the d i statistic from adjacent chromosomes. The dashed red line denotes the 99th percentile for each breed. [file 12864_2015_1384_MOESM9_ESM.png]
