# Supplementary material for: Genome-wide analysis reveals population structure and selection in Chinese indigenous sheep breeds
Source: BMC Genomics. 2015 Mar 17;16(1):194. doi: 10.1186/s12864-015-1384-9 (PMC4404018; doi:10.1186/s12864-015-1384-9)
Supplement: Additional file 3: Table S2. — The average distance between SNPs of different r2 values. [file 12864_2015_1384_MOESM3_ESM.docx]

**Table S2. The average distance between SNPs of different r^2^ values**

| Breed | Average distance between SNP(kb) | | | | | |
| --- | --- | --- | --- | --- | --- | --- |
|  | r^2^=0.1 | r^2^=0.2 | r^2^=0.3 | r^2^=0.4 | r^2^=0.5 | r^2^=0.6 |
| UJI | 233.12 | 223.97 | 228.22 | 208.45 | 184.95 | 173.54 |
| HUS | 236.48 | 225.05 | 221.79 | 199.04 | 173.35 | 188.10 |
| TON | 260.11 | 205.57 | 193.88 | 178.53 | 207.15 | 174.69 |
| LTH | 225.40 | 222.68 | 219.50 | 173.81 | 192.02 | 139.33 |
| LOP | 230.55 | 237.56 | 222.18 | 214.72 | 172.28 | 123.58 |
| KAZ | 233.47 | 225.76 | 217.90 | 205.57 | 178.80 | 123.55 |
| DUL | 241.03 | 237.92 | 219.45 | 228.80 | 159.00 | 147.46 |
| DQS | 239.44 | 234.42 | 220.06 | 209.02 | 154.69 | 136.34 |
| TIBP | 233.15 | 224.56 | 208.31 | 190.75 | 166.05 | 178.75 |
| TIBS | 229.84 | 229.64 | 215.53 | 197.80 | 203.15 | 165.73 |
| Average(SD) | 236.26(9.55) | 226.71(9.37) | 216.68(9.48) | 200.65(16.55) | 179.14(17.67) | 155.11(23.88) |
