# Supplementary material for: Genome-wide analysis reveals population structure and selection in Chinese indigenous sheep breeds
Source: BMC Genomics. 2015 Mar 17;16(1):194. doi: 10.1186/s12864-015-1384-9 (PMC4404018; doi:10.1186/s12864-015-1384-9)
Supplement: Additional file 5: Table S3. — Pairwise F ST among 10 Chinese indigenous breeds. [file 12864_2015_1384_MOESM5_ESM.docx]

**Table S3. Pairwise *F_ST_* among 10 Chinese indigenous breeds**

| **Breed** | **HUS** | **TON** | **LTH** | **LOP** | **KAZ** | **DUL** | **DIQ** | **TIBP** | **TIBV** | **Mean pairwise Fst** |
| --- | --- | --- | --- | --- | --- | --- | --- | --- | --- | --- |
| **UJI** | 0.032 | 0.024 | 0.025 | 0.015 | 0.038 | 0.050 | 0.053 | 0.022 | 0.034 | 0.033 |
| **HUS** |  | 0.041 | 0.041 | 0.033 | 0.055 | 0.067 | 0.069 | 0.039 | 0.05 | 0.047 |
| **TON** |  |  | 0.032 | 0.026 | 0.048 | 0.060 | 0.06 | 0.032 | 0.044 | 0.041 |
| **LTH** |  |  |  | 0.026 | 0.048 | 0.060 | 0.061 | 0.032 | 0.043 | 0.041 |
| **LOP** |  |  |  |  | 0.035 | 0.044 | 0.056 | 0.026 | 0.038 | 0.033 |
| **KAZ** |  |  |  |  |  | 0.069 | 0.078 | 0.049 | 0.062 | 0.054 |
| **DUL** |  |  |  |  |  |  | 0.09 | 0.059 | 0.072 | 0.063 |
| **DIQ** |  |  |  |  |  |  |  | 0.039 | 0.046 | 0.061 |
| **TIBP** |  |  |  |  |  |  |  |  | 0.012 | 0.034 |
| **TIBV** |  |  |  |  |  |  |  |  |  | 0.045 |
