# Supplementary material for: Genome-wide analysis reveals population structure and selection in Chinese indigenous sheep breeds
Source: BMC Genomics. 2015 Mar 17;16(1):194. doi: 10.1186/s12864-015-1384-9 (PMC4404018; doi:10.1186/s12864-015-1384-9)
Supplement: Additional file 8: Table S4. — Details of the 599 candidate selection regions. [file 12864_2015_1384_MOESM8_ESM.docx]

**Table S4 Details of the 599 candidate selection regions**

Bold fonts indicate d values in the top 1%, of the empirical distribution of di for each breed. Breed abbreviations are described in Table 1.

| Chr | di window region(bp) | UJI | HUS | TON | LTH | LOP | KAZ | DUL | DIQ | TIBP | TIBV |
| --- | --- | --- | --- | --- | --- | --- | --- | --- | --- | --- | --- |
| 1 | 600000-900000 | **11.78** | 1.58 | 5.99 | **10.01** | 1.58 | **9.09** | 4.66 | 1.36 | 5.35 | 7.43 |
| 1 | 7200000-7500000 | 3.41 | 1.28 | 5.74 | -0.29 | 1.21 | 2.17 | 5.93 | 7.49 | **8.43** | 2.27 |
| 1 | 9600000-9900000 | 3.24 | 2.46 | 2.51 | 1.57 | 3.28 | 6.01 | 1.47 | 6.20 | **10.86** | 8.47 |
| 1 | 14100000-14400000 | -0.94 | -2.73 | -0.44 | -3.14 | -0.47 | -1.90 | -3.38 | **18.58** | -0.77 | -1.65 |
| 1 | 14700000-15000000 | -0.03 | **10.02** | 0.65 | -1.98 | -0.43 | -0.11 | 1.99 | 7.58 | 1.30 | 0.05 |
| 1 | 19200000-19500000 | -1.15 | 6.81 | -0.03 | 2.81 | 4.09 | 1.13 | -0.50 | **9.26** | 0.26 | -2.50 |
| 1 | 20100000-20400000 | -0.86 | **13.35** | 4.03 | -1.39 | 1.24 | -0.50 | -2.07 | 7.70 | -0.51 | -0.28 |
| 1 | 29700000-30000000 | 1.65 | **8.71** | 2.87 | 5.15 | 1.10 | 1.34 | 0.91 | **19.18** | -0.07 | 4.93 |
| 1 | 36900000-37200000 | -3.34 | 2.32 | **10.22** | -2.37 | -1.35 | 2.50 | -3.26 | 1.78 | **7.99** | 1.07 |
| 1 | 42000000-42300000 | 0.71 | -2.96 | 3.94 | -1.47 | -1.98 | **8.59** | -2.17 | -0.02 | -0.21 | 0.60 |
| 1 | 44400000-44700000 | 5.55 | 3.82 | **9.01** | 2.02 | 2.14 | -0.83 | 9.44 | -0.30 | 3.52 | 5.30 |
| 1 | 47100000-47400000 | 0.62 | 3.18 | **8.27** | 6.10 | 0.71 | 3.62 | 0.27 | 0.98 | 6.89 | -0.95 |
| 1 | 48600000-48900000 | 1.41 | 1.27 | 0.48 | 2.77 | **7.91** | -0.26 | -1.50 | 1.36 | 2.66 | 0.89 |
| 1 | 55200000-55500000 | -0.12 | -2.75 | **11.59** | -1.54 | -0.42 | -0.50 | 0.45 | -0.73 | -0.62 | 1.14 |
| 1 | 60300000-60600000 | 2.49 | 5.94 | **8.77** | 4.45 | 2.83 | **9.41** | 7.11 | 0.92 | **9.92** | 2.64 |
| 1 | 66600000-66900000 | -1.03 | **11.25** | 1.21 | 1.69 | -0.02 | 3.88 | 0.77 | 0.23 | 0.35 | 0.79 |
| 1 | 66900000-67200000 | 7.12 | 1.67 | 0.49 | 1.52 | 3.86 | **8.47** | -0.55 | 4.78 | 5.70 | -0.80 |
| 1 | 68100000-68400000 | 0.92 | 1.17 | 0.96 | 3.36 | 1.96 | 3.77 | 8.30 | **9.37** | 3.24 | 2.49 |
| 1 | 69000000-69300000 | -1.24 | -0.85 | 0.43 | 0.09 | 4.70 | **11.48** | 1.51 | -1.49 | -0.48 | 1.20 |
| 1 | 76800000-77100000 | 2.07 | **8.69** | 2.71 | 4.55 | 1.89 | 1.29 | 4.88 | 5.97 | 3.66 | -0.56 |
| 1 | 89700000-90000000 | -2.25 | **17.81** | -1.97 | -1.38 | -2.00 | -0.24 | -3.46 | -3.15 | 0.04 | -2.18 |
| 1 | 99300000-99600000 | 1.52 | -0.44 | 0.32 | -2.89 | -1.29 | -2.59 | -2.90 | -0.45 | **8.19** | 0.42 |
| 1 | 101400000-101700000 | -0.31 | 0.87 | 1.76 | -0.58 | **7.81** | 3.39 | 2.39 | 4.95 | 3.63 | 0.40 |
| 1 | 105600000-105900000 | -1.84 | -3.50 | -2.58 | 0.82 | 2.21 | -2.52 | -1.22 | **12.16** | -3.44 | -2.92 |
| 1 | 115200000-115500000 | 5.84 | 0.83 | 4.73 | 2.81 | 3.88 | 5.73 | 1.34 | 1.95 | 5.14 | **11.95** |
| 1 | 117300000-117600000 | 1.59 | 1.50 | 1.40 | 3.73 | **13.51** | 5.48 | 7.50 | 6.96 | 1.36 | 0.11 |
| 1 | 120300000-120600000 | 1.30 | -1.37 | 5.97 | 2.70 | 5.82 | 4.17 | -1.48 | -0.27 | -0.22 | **10.45** |
| 1 | 123300000-123600000 | 1.31 | -2.03 | -0.02 | 0.96 | **8.94** | 2.61 | -1.94 | -2.70 | -1.24 | 0.90 |
| 1 | 130800000-131100000 | 2.10 | 4.31 | **11.89** | 5.30 | -0.24 | **8.14** | -0.08 | -0.15 | 3.00 | -0.83 |
| 1 | 132300000-132600000 | 7.05 | 4.30 | 2.63 | -1.22 | **11.39** | -0.92 | -1.09 | -0.45 | 0.36 | -0.08 |
| 1 | 145500000-145800000 | 0.94 | 3.03 | -0.68 | 0.03 | **8.55** | 1.29 | 1.73 | -0.78 | -0.30 | -0.28 |
| 1 | 162900000-163200000 | 3.49 | **10.46** | 5.27 | -0.25 | 2.19 | 4.46 | 0.41 | 6.30 | 0.03 | **9.99** |
| 1 | 163800000-164100000 | -0.35 | 1.72 | -0.32 | 1.77 | 1.61 | -0.65 | **10.97** | 1.40 | -1.90 | -3.40 |
| 1 | 166500000-166800000 | -0.03 | **10.13** | 4.48 | 3.41 | 2.27 | -1.43 | 0.15 | -1.05 | 1.17 | 1.51 |
| 1 | 177000000-177300000 | 0.72 | 1.89 | 1.68 | -0.32 | 2.18 | **10.33** | 2.73 | 3.24 | 6.32 | 1.65 |
| 1 | 183600000-183900000 | 0.29 | 2.80 | 0.96 | 3.10 | 0.43 | 1.45 | 0.91 | **11.71** | 3.62 | 3.94 |
| 1 | 191100000-191400000 | 4.59 | 6.46 | 3.46 | 8.48 | **10.05** | 6.58 | 8.58 | 1.60 | 3.28 | **9.67** |
| 1 | 191700000-192000000 | 0.13 | 4.45 | 2.73 | 1.46 | 1.08 | -0.65 | -2.13 | **10.71** | 1.66 | 1.14 |
| 1 | 195900000-196200000 | 5.07 | 1.68 | -1.07 | -0.25 | 3.14 | 2.70 | 0.90 | **13.67** | -1.22 | 0.20 |
| 1 | 199500000-199800000 | -0.97 | 5.41 | -0.24 | 2.84 | **8.77** | 3.02 | -1.21 | -2.08 | 0.78 | 3.51 |
| 1 | 204300000-204600000 | **11.98** | 0.47 | -0.24 | 0.62 | 1.87 | -0.13 | 2.09 | 1.41 | 3.43 | 2.03 |
| 1 | 214500000-214800000 | 6.25 | -0.07 | 0.26 | 1.63 | 1.76 | -0.25 | -2.27 | 6.96 | **11.17** | 1.78 |
| 1 | 216000000-216300000 | -0.61 | 3.76 | -0.42 | -2.20 | -0.09 | **12.82** | -2.39 | -1.04 | -0.01 | -1.62 |
| 1 | 225900000-226200000 | -0.05 | 3.48 | **8.31** | 1.37 | 2.71 | 0.06 | 2.16 | 1.47 | -1.42 | 1.09 |
| 1 | 228000000-228300000 | 5.18 | 0.51 | 2.46 | 2.15 | 1.01 | **12.45** | 1.29 | 6.60 | 4.04 | 3.85 |
| 1 | 230700000-231000000 | 4.14 | 1.70 | 5.86 | **9.11** | 6.95 | 6.45 | 4.49 | 8.72 | 4.65 | **12.57** |
| 1 | 234600000-234900000 | 2.53 | **10.31** | 5.02 | 7.66 | 0.84 | 7.71 | 2.99 | 0.20 | **7.81** | -0.96 |
| 1 | 236100000-236400000 | -1.24 | -0.63 | 1.67 | 1.65 | 0.10 | **8.70** | 3.80 | -1.35 | 0.16 | -1.44 |
| 1 | 240000000-240300000 | 1.25 | 7.34 | -1.08 | -1.11 | **8.64** | -0.02 | -1.02 | 1.90 | -0.49 | -1.94 |
| 1 | 243300000-243600000 | -0.14 | 4.53 | 2.12 | 6.10 | 4.26 | **9.23** | 1.28 | 7.97 | 1.67 | 0.03 |
| 1 | 244800000-245100000 | 2.15 | -1.42 | -1.11 | 0.60 | -1.71 | 5.25 | -0.49 | -1.61 | **10.17** | 2.57 |
| 1 | 245400000-245700000 | 0.59 | -1.75 | -0.09 | 0.00 | -1.15 | 0.04 | 0.03 | 2.31 | **7.65** | 0.51 |
| 1 | 247800000-248100000 | -0.88 | **11.82** | 6.36 | 0.40 | 1.65 | -0.41 | 1.62 | -2.57 | 4.51 | -1.46 |
| 1 | 248100000-248400000 | -0.28 | -1.27 | 0.42 | 4.77 | **7.81** | **9.38** | -0.13 | 8.44 | 1.88 | -0.87 |
| 1 | 254400000-254700000 | 1.19 | -2.60 | 0.40 | **9.14** | 0.91 | -0.38 | -1.72 | 4.16 | -1.32 | 0.94 |
| 1 | 255600000-255900000 | -1.69 | 0.36 | -2.19 | -1.34 | 6.15 | -0.47 | -3.44 | **9.22** | -0.06 | -3.29 |
| 1 | 264300000-264600000 | 0.04 | 5.95 | 3.80 | -0.71 | 0.98 | **8.59** | -0.27 | 0.92 | 0.37 | 1.38 |
| 2 | 3000000-3300000 | **8.54** | 0.65 | 4.52 | 1.44 | 4.16 | 2.73 | 7.14 | 2.62 | 2.86 | **9.61** |
| 2 | 6300000-6600000 | **8.62** | -0.30 | -0.13 | -0.37 | 3.75 | 3.10 | 1.32 | -1.29 | -0.76 | 1.24 |
| 2 | 9600000-9900000 | -1.97 | 0.86 | -1.14 | 3.27 | 0.27 | **8.54** | -1.56 | -0.45 | -0.31 | 2.02 |
| 2 | 11100000-11400000 | 1.41 | 1.56 | 1.75 | 0.75 | 4.44 | **10.13** | 3.63 | -1.40 | -0.29 | -0.89 |
| 2 | 11700000-12000000 | 7.43 | 3.73 | 5.04 | 4.04 | 2.01 | 1.76 | -0.38 | 1.19 | 0.65 | **9.33** |
| 2 | 12000000-12300000 | 2.62 | 7.03 | 6.46 | **11.93** | 4.80 | 3.51 | 0.07 | 1.88 | 1.98 | -0.26 |
| 2 | 15300000-15600000 | 0.25 | -0.39 | -0.42 | 0.70 | 4.36 | 0.89 | -0.69 | 6.71 | 1.60 | **10.63** |
| 2 | 16500000-16800000 | -2.09 | -0.67 | -1.35 | -3.13 | 1.40 | **8.78** | -1.62 | 0.25 | -2.06 | -2.75 |
| 2 | 27000000-27300000 | 1.27 | **15.83** | 1.28 | 0.47 | 4.66 | 2.14 | -1.71 | 1.69 | -0.45 | 3.28 |
| 2 | 28800000-29100000 | 3.56 | -3.92 | -4.17 | -3.62 | -2.44 | -3.78 | 2.99 | -4.95 | **7.64** | -2.26 |
| 2 | 30300000-30600000 | 5.98 | 4.48 | 2.79 | 1.95 | 3.67 | 5.33 | **19.31** | 3.06 | **11.68** | **13.30** |
| 2 | 32400000-32700000 | 1.86 | -2.50 | -1.85 | **12.47** | -1.29 | 1.51 | 0.93 | 0.30 | 0.53 | -2.08 |
| 2 | 35100000-35400000 | -1.46 | -1.42 | -1.11 | 1.61 | **8.96** | 1.43 | -1.76 | 4.37 | -0.01 | 0.14 |
| 2 | 35700000-36000000 | 0.79 | **14.44** | 4.04 | 0.35 | -0.01 | 0.57 | **10.77** | 5.91 | -0.03 | 2.39 |
| 2 | 37800000-38100000 | 1.51 | 2.72 | **7.93** | 0.43 | 0.21 | 1.75 | 5.62 | 8.11 | 0.69 | -1.40 |
| 2 | 42900000-43200000 | -0.78 | 2.24 | 3.49 | 0.43 | 1.22 | -1.92 | **11.41** | -0.57 | -1.45 | 1.20 |
| 2 | 50400000-50700000 | 6.49 | 0.88 | **12.40** | **9.39** | 0.17 | -0.23 | 4.26 | 3.93 | 3.85 | **13.89** |
| 2 | 51300000-51600000 | -1.56 | -0.68 | 2.46 | -0.12 | -1.60 | -1.06 | **10.90** | 0.76 | 0.37 | 2.62 |
| 2 | 51600000-51900000 | 2.70 | -0.31 | 4.76 | 3.39 | 2.86 | 1.82 | **14.77** | 1.05 | 3.31 | **9.29** |
| 2 | 51900000-52200000 | 7.38 | 1.88 | 2.75 | 3.39 | 1.00 | 2.81 | **19.02** | 1.38 | 0.79 | 2.77 |
| 2 | 52200000-52500000 | 5.39 | 5.40 | 5.35 | 2.90 | 5.54 | 4.23 | **32.23** | 2.97 | **7.85** | **10.15** |
| 2 | 52500000-52800000 | 1.03 | 1.24 | 2.56 | 0.88 | 3.37 | 2.75 | **27.68** | 3.99 | 3.62 | 1.32 |
| 2 | 52800000-53100000 | -0.01 | 0.39 | 7.00 | -0.31 | **8.12** | -1.33 | **18.17** | 3.05 | 4.98 | 1.00 |
| 2 | 53100000-53400000 | 4.29 | 2.08 | 2.05 | 0.49 | 2.57 | 0.88 | **31.47** | 6.14 | 2.68 | 3.03 |
| 2 | 53400000-53700000 | **11.67** | 5.00 | 4.44 | 1.95 | 4.03 | 3.26 | **27.25** | 2.18 | 5.78 | 5.78 |
| 2 | 53700000-54000000 | 0.95 | 1.33 | 0.62 | 2.48 | 5.12 | 0.56 | **16.63** | -1.88 | 1.16 | 1.43 |
| 2 | 54000000-54300000 | 2.33 | -0.35 | 2.24 | -1.07 | -0.29 | 1.30 | **26.02** | 0.00 | 4.33 | -0.28 |
| 2 | 54300000-54600000 | 5.59 | 1.92 | 2.58 | 0.18 | 1.55 | 4.88 | **21.06** | 4.66 | 1.83 | 4.49 |
| 2 | 54600000-54900000 | 2.01 | 3.29 | 3.69 | 4.41 | 1.90 | 1.29 | **23.73** | 2.78 | 1.63 | 0.07 |
| 2 | 54900000-55200000 | 1.93 | 1.22 | 4.03 | 3.61 | 6.15 | 2.99 | **33.69** | 0.84 | 4.36 | 2.90 |
| 2 | 55200000-55500000 | 1.35 | **9.40** | 1.89 | 3.54 | 2.10 | -0.33 | **29.78** | -0.04 | 0.19 | 4.25 |
| 2 | 55500000-55800000 | 1.94 | 4.09 | 3.78 | 3.07 | 1.15 | 1.36 | **26.94** | -0.47 | 0.95 | 0.70 |
| 2 | 55800000-56100000 | -0.37 | -0.49 | 2.66 | -0.88 | 2.09 | 2.45 | **11.84** | 1.47 | 3.42 | 0.44 |
| 2 | 56400000-56700000 | 1.05 | 3.23 | 4.70 | 0.49 | 5.37 | 3.14 | **17.57** | 1.35 | 3.84 | 0.08 |
| 2 | 57000000-57300000 | -1.54 | -0.03 | 0.84 | -1.37 | -0.48 | 0.51 | **19.55** | 4.65 | 3.16 | -0.24 |
| 2 | 57600000-57900000 | -0.47 | -0.86 | 1.58 | -1.01 | 4.60 | 2.05 | **11.54** | 0.25 | 2.06 | 3.95 |
| 2 | 58200000-58500000 | 1.32 | 7.66 | 2.08 | 1.69 | 5.17 | 5.07 | **11.71** | 5.57 | 6.15 | 6.22 |
| 2 | 58500000-58800000 | 3.17 | 2.48 | 2.84 | 5.12 | 5.22 | 3.29 | **16.38** | 1.86 | 3.71 | 4.53 |
| 2 | 58800000-59100000 | 6.43 | 0.64 | 0.48 | -0.17 | **14.32** | 1.08 | 7.59 | 0.69 | 2.58 | 1.68 |
| 2 | 59100000-59400000 | 0.96 | -0.27 | 2.04 | 5.19 | 4.30 | 0.85 | **11.42** | -0.96 | 2.76 | -0.16 |
| 2 | 62400000-62700000 | -0.89 | -0.79 | -0.34 | -1.47 | 2.55 | 0.96 | -2.54 | -2.20 | 4.90 | **9.13** |
| 2 | 69000000-69300000 | 0.54 | 1.27 | 3.25 | -0.97 | -0.08 | -0.58 | 1.61 | **12.47** | -0.28 | 2.97 |
| 2 | 75900000-76200000 | 0.23 | -2.06 | -1.41 | 0.57 | 1.46 | -2.03 | -0.78 | **10.41** | -2.71 | -1.02 |
| 2 | 81300000-81600000 | 0.96 | 4.43 | 5.23 | **17.25** | 2.73 | 2.71 | 1.88 | 1.48 | 1.50 | 0.64 |
| 2 | 81600000-81900000 | 3.50 | 1.93 | **10.28** | **16.02** | **8.01** | 6.12 | 1.45 | 1.23 | **10.81** | 7.66 |
| 2 | 81900000-82200000 | 5.33 | 4.41 | 5.43 | **17.80** | 5.51 | 1.63 | 0.62 | 5.10 | 2.91 | 6.87 |
| 2 | 89700000-90000000 | -2.16 | -0.11 | 1.39 | 1.28 | -0.88 | **9.14** | 2.59 | 6.32 | -0.92 | -0.73 |
| 2 | 90000000-90300000 | -0.29 | -0.40 | -2.51 | -2.44 | 0.54 | -0.57 | **11.40** | -0.50 | 3.98 | 1.70 |
| 2 | 107400000-107700000 | -1.60 | **9.74** | -1.71 | 0.87 | -2.39 | 0.02 | -1.88 | -0.98 | 1.31 | 3.60 |
| 2 | 110700000-111000000 | **9.31** | 0.55 | 3.34 | 4.80 | 3.62 | 0.04 | -0.38 | 3.79 | 1.78 | 6.10 |
| 2 | 112500000-112800000 | **10.11** | 5.93 | 2.47 | 5.82 | **10.46** | 1.14 | -0.89 | 0.17 | 6.16 | **11.86** |
| 2 | 113100000-113400000 | **8.85** | -2.72 | -3.30 | -3.16 | -3.14 | 1.47 | -1.76 | -3.61 | 0.57 | -1.51 |
| 2 | 114300000-114600000 | **15.83** | -0.18 | 5.89 | -1.43 | 0.45 | 3.95 | -1.90 | -1.81 | -0.52 | 4.17 |
| 2 | 122100000-122400000 | 2.59 | 8.57 | 3.47 | 3.74 | **7.56** | 2.95 | 9.61 | 0.07 | 0.58 | 3.16 |
| 2 | 122400000-122700000 | 1.47 | **10.21** | 4.83 | 5.14 | **14.32** | 3.74 | **16.36** | 3.39 | 3.28 | 1.68 |
| 2 | 123000000-123300000 | **9.99** | 0.69 | 3.10 | -1.33 | 0.91 | 4.78 | 0.16 | 5.60 | 2.37 | 5.08 |
| 2 | 125400000-125700000 | 1.64 | 5.09 | 4.77 | **9.61** | 1.05 | -0.83 | 0.48 | 6.22 | 0.32 | 0.72 |
| 2 | 134400000-134700000 | 1.39 | 1.01 | 2.90 | 3.08 | 1.61 | -0.17 | -2.12 | 0.53 | 0.79 | **11.60** |
| 2 | 135300000-135600000 | 2.36 | 1.83 | -0.33 | -0.27 | 0.54 | 2.09 | 2.01 | 0.09 | **7.74** | 5.98 |
| 2 | 147000000-147300000 | 3.00 | 5.22 | **7.29** | 1.89 | 0.87 | 0.72 | -0.37 | -1.98 | 5.13 | 3.96 |
| 2 | 159000000-159300000 | 4.88 | 1.33 | 2.34 | 2.49 | **8.03** | 1.10 | -0.81 | 0.32 | 5.92 | 5.49 |
| 2 | 166800000-167100000 | -1.14 | -1.71 | -0.18 | 0.81 | 1.95 | **8.78** | -1.95 | -1.87 | 0.38 | 2.45 |
| 2 | 173700000-174000000 | -0.99 | **9.35** | 2.35 | 2.65 | 5.77 | -0.20 | -1.80 | -2.93 | 1.47 | 2.55 |
| 2 | 175500000-175800000 | -0.98 | **11.33** | -1.70 | -0.93 | -2.12 | -0.61 | -1.82 | 0.18 | -1.93 | -0.98 |
| 2 | 177300000-177600000 | -0.08 | -1.79 | **8.00** | -2.28 | 0.03 | -0.21 | -2.27 | -0.14 | -1.31 | -1.98 |
| 2 | 180000000-180300000 | -2.54 | -2.18 | -1.80 | -0.77 | -1.21 | 4.25 | 1.93 | 2.71 | **8.07** | 2.39 |
| 2 | 180600000-180900000 | -0.29 | -1.37 | -0.46 | -3.01 | 1.31 | 1.91 | -2.30 | **14.70** | -0.63 | 1.00 |
| 2 | 181800000-182100000 | 0.17 | -0.68 | -0.07 | -0.09 | **8.15** | 1.30 | 0.39 | 5.46 | 0.55 | 0.26 |
| 2 | 182400000-182700000 | 0.90 | -0.66 | 2.91 | 5.55 | 2.37 | 0.52 | 1.19 | **14.11** | 3.04 | **8.96** |
| 2 | 183900000-184200000 | -0.82 | **9.10** | -0.07 | -1.22 | -2.28 | -0.38 | -1.62 | -0.02 | -0.17 | 0.83 |
| 2 | 189300000-189600000 | 6.59 | 3.47 | 6.06 | **10.95** | 1.56 | 3.85 | 0.74 | 2.60 | 0.69 | 2.59 |
| 2 | 189600000-189900000 | -1.61 | -1.24 | 0.45 | 1.83 | 1.83 | 0.05 | **11.86** | -1.85 | -0.13 | 1.21 |
| 2 | 196500000-196800000 | 3.28 | 3.55 | 0.64 | -0.86 | **11.09** | 4.00 | 0.19 | 1.31 | -0.97 | 0.57 |
| 2 | 197100000-197400000 | 4.83 | 7.42 | 4.15 | 7.74 | **7.78** | 3.12 | 1.90 | 4.91 | 1.60 | **12.29** |
| 2 | 198000000-198300000 | -1.44 | **11.92** | 1.16 | 4.84 | -0.64 | 5.62 | -0.28 | 0.70 | -0.64 | 0.34 |
| 2 | 200100000-200400000 | **15.25** | 1.43 | 4.55 | 6.67 | 3.61 | 0.84 | 6.44 | 2.47 | 0.23 | 4.04 |
| 2 | 202500000-202800000 | 0.50 | 3.47 | 1.63 | 4.36 | 0.42 | 1.45 | -0.50 | 0.41 | 0.29 | **9.16** |
| 2 | 208800000-209100000 | -1.04 | 2.49 | 0.72 | **11.06** | 1.42 | 2.69 | -2.26 | 0.00 | -0.15 | 1.39 |
| 2 | 209100000-209400000 | 3.18 | **10.90** | -0.31 | 4.19 | -0.51 | -1.49 | -1.80 | 1.95 | 0.09 | 4.74 |
| 2 | 218100000-218400000 | 7.33 | 3.71 | 5.63 | **14.11** | 2.71 | 7.73 | **16.44** | 2.01 | 6.68 | 8.35 |
| 2 | 218700000-219000000 | **12.07** | -2.64 | -1.05 | 5.07 | -0.29 | 3.11 | 10.39 | -1.85 | -0.57 | 4.50 |
| 2 | 219000000-219300000 | **16.21** | 1.76 | 4.77 | 5.01 | 2.11 | 6.70 | 8.16 | 1.07 | **8.38** | 7.04 |
| 2 | 219600000-219900000 | -0.31 | 5.58 | 1.68 | 8.00 | 1.68 | **10.01** | **14.70** | 2.08 | 1.09 | 1.87 |
| 2 | 222000000-222300000 | 2.76 | **12.67** | -0.59 | -0.32 | 1.00 | 0.81 | 0.61 | 1.14 | -0.40 | 3.46 |
| 2 | 228600000-228900000 | **8.52** | 3.49 | 4.63 | 1.21 | 0.03 | 2.20 | **10.67** | -0.15 | -0.76 | 1.59 |
| 2 | 232500000-232800000 | -0.18 | -1.18 | -2.02 | **8.60** | -1.46 | 1.59 | -0.76 | -1.07 | -0.34 | 4.00 |
| 2 | 232800000-233100000 | **9.05** | 2.16 | 5.73 | 7.85 | 3.53 | 6.67 | 0.79 | **14.81** | 5.71 | 3.88 |
| 3 | 6600000-6900000 | **8.19** | 6.74 | -0.14 | 1.13 | 1.12 | 0.97 | -1.38 | -0.22 | 0.92 | -0.15 |
| 3 | 9900000-10200000 | -1.90 | 5.44 | **7.85** | 1.32 | 0.91 | -1.82 | 0.29 | 0.97 | -0.70 | -0.02 |
| 3 | 11700000-12000000 | **9.31** | 3.22 | 0.97 | 0.72 | -0.71 | -2.39 | 0.61 | -2.93 | -0.61 | -0.86 |
| 3 | 13800000-14100000 | 6.63 | -1.06 | 1.02 | 0.24 | 0.34 | 0.28 | -0.57 | **12.34** | 3.41 | 1.15 |
| 3 | 23700000-24000000 | 1.20 | 7.58 | 1.27 | -0.92 | 1.29 | 1.76 | **12.22** | -0.09 | 2.63 | -0.49 |
| 3 | 30900000-31200000 | 2.54 | 1.72 | -0.13 | -0.29 | 0.91 | 1.94 | **10.91** | 3.25 | 2.84 | 0.21 |
| 3 | 31500000-31800000 | **9.05** | 1.52 | -0.54 | 1.01 | **8.58** | 0.83 | **11.86** | -1.74 | -1.08 | 1.21 |
| 3 | 39600000-39900000 | 4.28 | 2.20 | 2.94 | 3.58 | 1.35 | 0.44 | 4.18 | 4.24 | **12.41** | 8.63 |
| 3 | 39900000-40200000 | **8.36** | 4.24 | 5.06 | 1.50 | 2.95 | 7.32 | 2.01 | 3.36 | **20.32** | 4.67 |
| 3 | 40200000-40500000 | 3.12 | 2.81 | 5.56 | 3.42 | 4.35 | 5.21 | 6.78 | 4.16 | **17.04** | **10.08** |
| 3 | 40500000-40800000 | 5.57 | 7.62 | 4.87 | **8.79** | 2.98 | 3.53 | 4.01 | **11.35** | **14.29** | 4.78 |
| 3 | 54900000-55200000 | -2.07 | -2.57 | 2.19 | **10.22** | 1.69 | -2.17 | -2.22 | -2.18 | 1.99 | -3.28 |
| 3 | 58500000-58800000 | -0.08 | -1.64 | 2.84 | **9.07** | -0.12 | -1.25 | 4.83 | 0.15 | 2.26 | 5.59 |
| 3 | 60300000-60600000 | 0.16 | -2.14 | -0.87 | 0.47 | **9.90** | 0.59 | -3.60 | 2.45 | -0.04 | -1.36 |
| 3 | 61800000-62100000 | 0.50 | 0.68 | **7.64** | -0.34 | -0.42 | 0.64 | -1.93 | 2.27 | **7.70** | 2.35 |
| 3 | 67800000-68100000 | 4.44 | -0.73 | **15.90** | 3.58 | 0.46 | 0.21 | 0.74 | 3.85 | 2.08 | 0.00 |
| 3 | 69000000-69300000 | 2.91 | 5.19 | 4.07 | 0.47 | 5.35 | 1.99 | 1.38 | 4.29 | **11.03** | 0.17 |
| 3 | 70800000-71100000 | -0.39 | -1.61 | 0.64 | -1.04 | 2.76 | **12.17** | 1.61 | 0.05 | -0.13 | 2.67 |
| 3 | 79500000-79800000 | 2.67 | 1.32 | **7.34** | 0.49 | 3.19 | 1.76 | -1.57 | 1.89 | 0.23 | 0.42 |
| 3 | 91500000-91800000 | -0.90 | 1.56 | 2.31 | -0.48 | -0.07 | **9.66** | 1.43 | 0.83 | 2.08 | -1.73 |
| 3 | 93300000-93600000 | 1.43 | 3.74 | 5.68 | 6.77 | 2.39 | 4.66 | -0.20 | 6.14 | 3.16 | **9.27** |
| 3 | 93600000-93900000 | 3.41 | 3.83 | 3.77 | **8.62** | 4.86 | 6.32 | 4.41 | 2.80 | **13.26** | **11.64** |
| 3 | 97800000-98100000 | -1.33 | -1.19 | 3.00 | **8.76** | 5.58 | 1.31 | 1.02 | 2.38 | 0.38 | **11.61** |
| 3 | 100500000-100800000 | 2.33 | **11.32** | 1.80 | -0.69 | 1.68 | 3.09 | **13.64** | 1.52 | 2.98 | 5.84 |
| 3 | 102900000-103200000 | -1.22 | 3.90 | 3.20 | **8.66** | 0.19 | 3.91 | -1.15 | 1.93 | -0.15 | 5.96 |
| 3 | 107100000-107400000 | 5.01 | 1.73 | 2.66 | 3.42 | 5.47 | 1.91 | 2.70 | 4.13 | **7.82** | **22.49** |
| 3 | 109200000-109500000 | **9.61** | 0.11 | -0.68 | -1.11 | 0.61 | -1.74 | 6.24 | -2.83 | -0.57 | 1.94 |
| 3 | 109500000-109800000 | **9.95** | 0.74 | -0.63 | 7.10 | 1.52 | -0.37 | 4.48 | -3.41 | 1.32 | 2.54 |
| 3 | 110400000-110700000 | 2.44 | 2.73 | 1.83 | 1.07 | 2.01 | 5.55 | 0.49 | 0.97 | **8.57** | 3.11 |
| 3 | 110700000-111000000 | 3.35 | 4.07 | -0.10 | **8.92** | 2.37 | 2.42 | 5.06 | 1.40 | 5.30 | -0.75 |
| 3 | 119400000-119700000 | -0.51 | 0.28 | -0.26 | -2.00 | **9.83** | 1.72 | 0.00 | -0.02 | -1.03 | -0.31 |
| 3 | 119700000-120000000 | -1.23 | -1.22 | -1.54 | 0.65 | **8.97** | -0.48 | -2.71 | 0.13 | -1.67 | -3.05 |
| 3 | 120000000-120300000 | 3.31 | -2.16 | -2.41 | **9.01** | 3.06 | -0.31 | -2.68 | -0.99 | 0.94 | 2.68 |
| 3 | 123000000-123300000 | 6.47 | 0.38 | 0.18 | -1.47 | **14.62** | 0.89 | -0.51 | 1.99 | -0.64 | 1.78 |
| 3 | 127500000-127800000 | **8.92** | -0.08 | -0.13 | 0.81 | 0.15 | 1.44 | 1.42 | 0.25 | 0.59 | 1.73 |
| 3 | 127800000-128100000 | 6.87 | 0.67 | **9.15** | 3.70 | 2.76 | 4.36 | 3.97 | 0.44 | 3.46 | 4.95 |
| 3 | 129600000-129900000 | **8.76** | 2.54 | 0.96 | 1.01 | 2.41 | 0.31 | 1.53 | -1.03 | 3.89 | **17.64** |
| 3 | 129900000-130200000 | **8.14** | 1.30 | -0.80 | 2.10 | 1.29 | 1.57 | 4.37 | -0.27 | -0.45 | 0.48 |
| 3 | 133500000-133800000 | **8.65** | 6.02 | 4.30 | 4.00 | 6.68 | 3.84 | **14.37** | 1.97 | 5.65 | 3.75 |
| 3 | 134100000-134400000 | **11.09** | 1.35 | -1.10 | -1.98 | -0.33 | 3.50 | -1.67 | 0.33 | -0.29 | -0.34 |
| 3 | 136200000-136500000 | 1.11 | 6.36 | 1.24 | 1.12 | 5.13 | 2.04 | 4.52 | **10.00** | 3.88 | **9.10** |
| 3 | 137100000-137400000 | -0.37 | 0.88 | 0.12 | 2.70 | 1.75 | **12.04** | 3.25 | 2.24 | 0.93 | 4.23 |
| 3 | 138300000-138600000 | 3.92 | 1.63 | 3.27 | 2.60 | **16.87** | 5.81 | 2.04 | 1.90 | **13.56** | 1.12 |
| 3 | 139500000-139800000 | **8.10** | 1.47 | 1.49 | 4.88 | 2.42 | -0.30 | 1.29 | 1.92 | 5.84 | 1.22 |
| 3 | 143700000-144000000 | **10.82** | -0.35 | 1.60 | 0.53 | 1.77 | -1.50 | 0.80 | -0.51 | 4.62 | 6.68 |
| 3 | 144000000-144300000 | 7.25 | 6.72 | 3.95 | **9.52** | 4.41 | **12.45** | 5.21 | 1.45 | 2.78 | -0.03 |
| 3 | 145500000-145800000 | 0.96 | **8.89** | -0.36 | -0.60 | 1.44 | 2.92 | -0.98 | -0.47 | -0.57 | 2.56 |
| 3 | 147300000-147600000 | -1.21 | **9.49** | 0.47 | -2.42 | -2.35 | -2.57 | -1.28 | -1.10 | 2.75 | -1.27 |
| 3 | 152400000-152700000 | **8.37** | 8.02 | 3.14 | 1.44 | 0.72 | -0.71 | 0.27 | -2.74 | -1.04 | -0.20 |
| 3 | 153900000-154200000 | -1.23 | -2.52 | 2.67 | -1.46 | 1.23 | **8.34** | 2.37 | 5.12 | -1.59 | -0.67 |
| 3 | 154200000-154500000 | **12.01** | 6.66 | **11.70** | **12.25** | **9.56** | 6.71 | **23.22** | 8.02 | 7.26 | **10.81** |
| 3 | 161400000-161700000 | -1.67 | 1.38 | -0.73 | -1.99 | **8.24** | 0.04 | 0.29 | -2.12 | 0.64 | -0.29 |
| 3 | 162000000-162300000 | 2.18 | 4.88 | 2.99 | **14.85** | 6.16 | 3.80 | 1.50 | 4.31 | 1.63 | 0.42 |
| 3 | 162300000-162600000 | -1.92 | 1.13 | **7.55** | 2.97 | -0.69 | 1.39 | -0.09 | -3.09 | -1.83 | -0.70 |
| 3 | 165900000-166200000 | 1.22 | -1.01 | 0.85 | **8.89** | 1.09 | -0.81 | 1.61 | 4.71 | 1.70 | 2.54 |
| 3 | 166800000-167100000 | **8.24** | -0.79 | 1.65 | -0.34 | -1.64 | 1.95 | -2.34 | 1.13 | -0.81 | -1.75 |
| 3 | 176700000-177000000 | 3.02 | -0.30 | **8.59** | -0.54 | 1.52 | -1.70 | 1.11 | 2.24 | 1.11 | 3.31 |
| 3 | 178500000-178800000 | 0.07 | -0.79 | -0.89 | -0.61 | 0.22 | -2.53 | 3.15 | **15.07** | 1.43 | 3.65 |
| 3 | 179400000-179700000 | -0.87 | -2.61 | -0.06 | -0.34 | -0.34 | 2.62 | -0.98 | -2.19 | **10.51** | 1.80 |
| 3 | 180900000-181200000 | **10.82** | 1.54 | -1.57 | -1.95 | 1.29 | -3.04 | 0.01 | 3.51 | -1.59 | -1.78 |
| 3 | 181200000-181500000 | **9.39** | 1.69 | -0.57 | 2.46 | 1.49 | 0.47 | 1.17 | 7.44 | 3.90 | -0.07 |
| 3 | 181500000-181800000 | 2.36 | 1.12 | -0.12 | 4.23 | 1.38 | 1.33 | -1.08 | 1.31 | **9.76** | 3.96 |
| 3 | 181800000-182100000 | -2.55 | -2.15 | 2.83 | 0.78 | -0.67 | 1.67 | 1.24 | -3.20 | **8.28** | -2.80 |
| 3 | 183900000-184200000 | -0.70 | -0.87 | -1.38 | **9.09** | 4.87 | 1.34 | 0.89 | -0.43 | 0.40 | -1.68 |
| 3 | 189000000-189300000 | -0.06 | -0.40 | -0.98 | 1.33 | -0.24 | **9.91** | 3.95 | 5.29 | 0.87 | 4.80 |
| 3 | 192900000-193200000 | 1.10 | -0.76 | **9.74** | 1.44 | -0.40 | 1.74 | 0.51 | -0.40 | 5.36 | 5.34 |
| 3 | 196500000-196800000 | -0.48 | 0.26 | **9.12** | -1.51 | -2.07 | 1.03 | 2.93 | -2.51 | -0.48 | -0.54 |
| 3 | 200700000-201000000 | 1.86 | 2.14 | 0.05 | -0.78 | **7.51** | 0.62 | 5.16 | -1.18 | 2.20 | -0.17 |
| 3 | 215100000-215400000 | 3.14 | 0.34 | **9.43** | -0.49 | 0.01 | -0.31 | 1.02 | 1.15 | 1.56 | 0.94 |
| 3 | 215400000-215700000 | 1.20 | 1.48 | -0.16 | 1.65 | **8.98** | 5.55 | 5.48 | 1.49 | **12.69** | 6.03 |
| 3 | 215700000-216000000 | 2.50 | 0.68 | 2.47 | 1.74 | **7.49** | -0.86 | 3.06 | 2.99 | 3.92 | 1.79 |
| 4 | 600000-900000 | -2.31 | 3.22 | 4.41 | 0.14 | -0.27 | -0.89 | -0.93 | **10.33** | 2.11 | 3.78 |
| 4 | 2400000-2700000 | -0.70 | -0.51 | **13.47** | 2.55 | -1.27 | -0.64 | -1.57 | -0.77 | -0.63 | -2.59 |
| 4 | 3300000-3600000 | 1.40 | 7.47 | 5.59 | **9.82** | 1.30 | 1.37 | 1.01 | 0.15 | 1.78 | -0.50 |
| 4 | 5100000-5400000 | 5.00 | 2.67 | 3.13 | 2.81 | 2.41 | 2.62 | 3.29 | **10.54** | **7.67** | 4.56 |
| 4 | 7200000-7500000 | 1.36 | **9.80** | -1.16 | 0.50 | 0.55 | 0.04 | -2.61 | -2.01 | -0.16 | -2.32 |
| 4 | 8100000-8400000 | 2.85 | -0.32 | **10.79** | 0.54 | 0.70 | 6.98 | 0.81 | 1.75 | 6.10 | 0.63 |
| 4 | 10500000-10800000 | 0.39 | 2.56 | **7.74** | 1.39 | 1.19 | 2.03 | 0.87 | 0.95 | 1.11 | 1.42 |
| 4 | 33600000-33900000 | 1.22 | **8.82** | 0.33 | 5.57 | 2.88 | -0.61 | 0.90 | 0.55 | -0.23 | 0.92 |
| 4 | 39900000-40200000 | -1.91 | 1.94 | 0.30 | -1.45 | -0.42 | 4.71 | 0.21 | **10.48** | -1.43 | -2.47 |
| 4 | 42000000-42300000 | 0.98 | **11.46** | -1.50 | -1.11 | -0.85 | 0.29 | -0.93 | -1.63 | -2.25 | -1.14 |
| 4 | 43800000-44100000 | -0.27 | **9.36** | 6.21 | 4.90 | 1.19 | 1.48 | 1.75 | 6.09 | 0.97 | 2.40 |
| 4 | 48600000-48900000 | 5.08 | 1.86 | 2.52 | 0.83 | 5.01 | **10.34** | 1.82 | 6.45 | 2.39 | 4.59 |
| 4 | 51600000-51900000 | -0.66 | -1.11 | -1.29 | 1.05 | 6.10 | -0.14 | -2.50 | 0.16 | **10.56** | 0.11 |
| 4 | 68400000-68700000 | 3.08 | 4.94 | 1.83 | 7.17 | 5.48 | 2.46 | 5.97 | 3.11 | 4.39 | **11.56** |
| 4 | 68700000-69000000 | 4.02 | 2.81 | 3.06 | **11.17** | **8.52** | 4.37 | **11.27** | 6.21 | 5.88 | **15.82** |
| 4 | 69600000-69900000 | 0.53 | 5.79 | **8.81** | 0.66 | 6.61 | **10.55** | 7.92 | 1.21 | 4.18 | 8.45 |
| 4 | 75000000-75300000 | 1.48 | 0.72 | 0.51 | -0.55 | 1.98 | -0.73 | 6.80 | 1.93 | **12.00** | 3.42 |
| 4 | 75900000-76200000 | 0.11 | 2.11 | 0.14 | 0.03 | -0.73 | 1.16 | 1.29 | -1.34 | **8.90** | 4.99 |
| 4 | 77400000-77700000 | **10.88** | 8.20 | -0.43 | -0.26 | -0.30 | 2.54 | 3.43 | 4.23 | -0.08 | -1.86 |
| 4 | 90600000-90900000 | 1.08 | 0.57 | 3.95 | 3.30 | **8.04** | 2.91 | -0.66 | 4.33 | 7.41 | 5.94 |
| 4 | 113100000-113400000 | 1.64 | 1.28 | 1.76 | -1.17 | 2.53 | 1.22 | 1.45 | **12.15** | 4.01 | 0.03 |
| 5 | 18300000-18600000 | 2.16 | -1.86 | 0.32 | 4.38 | 3.69 | 3.41 | 6.44 | 2.73 | 0.41 | **9.10** |
| 5 | 19800000-20100000 | 2.49 | -0.61 | 0.59 | **14.12** | 1.46 | 0.66 | -2.17 | -0.19 | -0.08 | 4.16 |
| 5 | 26700000-27000000 | 0.85 | -2.73 | **7.58** | -2.23 | -1.72 | 0.99 | -2.86 | 2.68 | -0.58 | 0.13 |
| 5 | 36600000-36900000 | 0.85 | -0.94 | -1.09 | 0.52 | 2.67 | **8.43** | 3.08 | -1.23 | 0.06 | 1.58 |
| 5 | 40200000-40500000 | 2.42 | 7.51 | 3.40 | 0.30 | -1.45 | -0.57 | 1.10 | 2.81 | **8.49** | 1.15 |
| 5 | 42300000-42600000 | -0.28 | 3.58 | 2.43 | 1.41 | 3.36 | 6.89 | **11.76** | 3.13 | 3.75 | 5.09 |
| 5 | 46500000-46800000 | 3.70 | 1.58 | 0.14 | **17.41** | 0.32 | 0.15 | 1.80 | -0.41 | **8.77** | 1.23 |
| 5 | 47100000-47400000 | 1.25 | 2.12 | **14.01** | -1.09 | 1.18 | -0.18 | 6.18 | 1.45 | 0.34 | 2.08 |
| 5 | 47400000-47700000 | -1.43 | -1.12 | 6.81 | **14.09** | -2.78 | -0.71 | 0.11 | -2.00 | -1.52 | 0.11 |
| 5 | 56700000-57000000 | -0.79 | 1.01 | -0.32 | -1.35 | 0.02 | **10.35** | 0.19 | 1.09 | -0.86 | -1.11 |
| 5 | 57000000-57300000 | 0.79 | 1.19 | 0.51 | 2.27 | **7.59** | 7.60 | 1.76 | 0.00 | 2.41 | 4.80 |
| 5 | 57600000-57900000 | -1.77 | 0.32 | -0.62 | **18.47** | 1.51 | 4.03 | -1.75 | 3.04 | 6.68 | 3.06 |
| 5 | 78600000-78900000 | **8.43** | 0.23 | 1.67 | -0.08 | 0.88 | -0.05 | -0.40 | 2.89 | 1.59 | -0.23 |
| 5 | 79200000-79500000 | 1.41 | **10.96** | **10.68** | 0.11 | 0.80 | 1.82 | 0.25 | -0.39 | 5.39 | **15.49** |
| 5 | 79500000-79800000 | -0.92 | -1.18 | -0.35 | -2.81 | -2.08 | -0.90 | -2.48 | 1.65 | 1.46 | **9.09** |
| 5 | 89700000-90000000 | 2.63 | -2.61 | **8.86** | -0.15 | -0.05 | -1.38 | -0.87 | -2.95 | -2.28 | -2.50 |
| 5 | 99300000-99600000 | -0.90 | -1.17 | **9.22** | 0.86 | -0.80 | -1.37 | 1.78 | -0.23 | -0.02 | 1.21 |
| 5 | 104700000-105000000 | 2.48 | 0.13 | 4.29 | 0.02 | 4.31 | -0.05 | 0.39 | **9.92** | 0.90 | 1.11 |
| 6 | 13800000-14100000 | **10.10** | **9.91** | 3.44 | 2.79 | 2.12 | 4.77 | -1.17 | 5.77 | -0.45 | 1.87 |
| 6 | 18900000-19200000 | **12.58** | 4.09 | 3.33 | -1.66 | 0.69 | 0.18 | -0.35 | -0.16 | 1.14 | 7.80 |
| 6 | 26100000-26400000 | 5.23 | -0.89 | 2.83 | 4.06 | 4.77 | 2.67 | **11.45** | 5.28 | 1.30 | 0.41 |
| 6 | 27600000-27900000 | 2.10 | **8.88** | 0.75 | 0.77 | 6.93 | 2.51 | 3.31 | 0.39 | 2.65 | 1.18 |
| 6 | 29100000-29400000 | 1.86 | **9.97** | 3.84 | 3.85 | 1.20 | 1.88 | 4.17 | 1.16 | 1.09 | 0.08 |
| 6 | 29400000-29700000 | -0.18 | 5.68 | 0.82 | **9.30** | 0.57 | 1.77 | 8.02 | 2.12 | 2.98 | 0.05 |
| 6 | 30000000-30300000 | 0.71 | **9.73** | 4.02 | 4.60 | 1.14 | 2.92 | **15.42** | 4.58 | 2.57 | 2.61 |
| 6 | 30300000-30600000 | 2.51 | 3.74 | 1.44 | 1.14 | 2.79 | 2.57 | **20.97** | 0.64 | 2.82 | 0.10 |
| 6 | 30600000-30900000 | -0.88 | 3.47 | 0.93 | -0.19 | 1.20 | 1.91 | **11.90** | -0.13 | 4.46 | -0.21 |
| 6 | 32100000-32400000 | -1.00 | -0.31 | -1.70 | 2.24 | 1.16 | -1.23 | **17.86** | -1.79 | -1.14 | -1.53 |
| 6 | 32400000-32700000 | 0.29 | 4.82 | 0.30 | 2.29 | 0.85 | 1.96 | **22.64** | 2.88 | 1.14 | -1.19 |
| 6 | 33000000-33300000 | -0.58 | 1.67 | 0.24 | 2.07 | -0.65 | 2.24 | **17.81** | **9.48** | **8.80** | 1.25 |
| 6 | 33300000-33600000 | 0.62 | 4.20 | -0.63 | 1.04 | 0.06 | 0.42 | **10.67** | 3.24 | 1.70 | 1.08 |
| 6 | 33600000-33900000 | 2.61 | 0.79 | 1.50 | 3.20 | -0.13 | 1.78 | **10.77** | 2.41 | 0.81 | 5.56 |
| 6 | 34500000-34800000 | 3.20 | 1.12 | 4.40 | 7.12 | 2.08 | 3.64 | 9.80 | **11.70** | 5.04 | 2.54 |
| 6 | 34800000-35100000 | 2.84 | 3.96 | 0.98 | 3.31 | 2.44 | 2.11 | 6.84 | -0.87 | 2.27 | **9.66** |
| 6 | 36600000-36900000 | 7.60 | 5.99 | 5.32 | 4.25 | 2.87 | 3.13 | **25.07** | 3.68 | 1.73 | 2.40 |
| 6 | 37200000-37500000 | -1.04 | 1.67 | 2.03 | 1.14 | 0.92 | 3.15 | **13.61** | 1.67 | 1.19 | 1.28 |
| 6 | 37500000-37800000 | 1.73 | 4.77 | 4.63 | 0.21 | 2.35 | 4.41 | **19.42** | **10.83** | 1.67 | 1.63 |
| 6 | 37800000-38100000 | 0.35 | 0.18 | 0.10 | -0.41 | 0.56 | 1.02 | **11.96** | 0.66 | -0.86 | -2.23 |
| 6 | 38100000-38400000 | 3.02 | 3.10 | 4.17 | 4.50 | 2.15 | 0.84 | **23.89** | 3.75 | 1.30 | 1.21 |
| 6 | 39600000-39900000 | -2.66 | 0.82 | 1.49 | -2.96 | -0.83 | 2.86 | **11.12** | -1.05 | -3.04 | -2.83 |
| 6 | 39900000-40200000 | 2.44 | 1.29 | 6.72 | 1.68 | 3.68 | **8.68** | 7.89 | 1.04 | 1.17 | 4.95 |
| 6 | 50100000-50400000 | -0.85 | **12.38** | -0.05 | -0.67 | -0.95 | -0.80 | 0.65 | 0.11 | -1.72 | 1.33 |
| 6 | 54600000-54900000 | -1.78 | **11.10** | -1.57 | -1.79 | 0.04 | 0.62 | -1.70 | 1.16 | -1.16 | 7.69 |
| 6 | 56100000-56400000 | **8.29** | 1.49 | -0.38 | -1.55 | 4.80 | -0.26 | -0.20 | 0.05 | 1.65 | -2.02 |
| 6 | 57900000-58200000 | -0.04 | **11.99** | 0.90 | -1.02 | 0.23 | -0.47 | 0.08 | -1.83 | 2.79 | 1.59 |
| 6 | 60600000-60900000 | 1.92 | 4.80 | 5.32 | 0.61 | **8.87** | 3.51 | 2.95 | 1.23 | 1.59 | 2.34 |
| 6 | 68700000-69000000 | 2.95 | 0.59 | 3.41 | -0.16 | 2.93 | 2.83 | 4.35 | **11.52** | 1.55 | 4.29 |
| 6 | 69900000-70200000 | 2.64 | 1.42 | 2.10 | 0.86 | 1.14 | **8.44** | **23.16** | 0.30 | 3.61 | 5.32 |
| 6 | 75300000-75600000 | 6.23 | 1.01 | 2.10 | 2.54 | **11.68** | -0.56 | 6.52 | 0.49 | 0.39 | -1.25 |
| 6 | 76200000-76500000 | 6.47 | 7.56 | 3.21 | 6.52 | 3.05 | 2.33 | -0.17 | **9.92** | 2.28 | 5.77 |
| 6 | 78300000-78600000 | 2.04 | -1.18 | 3.09 | -2.58 | **14.85** | -2.98 | -1.37 | -2.34 | 0.74 | -1.81 |
| 6 | 80700000-81000000 | -0.02 | -0.27 | -0.84 | 0.04 | **7.88** | -1.88 | -1.37 | 2.08 | 1.95 | -2.82 |
| 6 | 85500000-85800000 | -1.08 | -0.96 | 4.42 | 4.34 | **10.09** | 4.22 | -1.76 | 0.57 | 0.73 | 4.23 |
| 6 | 111600000-111900000 | -0.30 | 1.45 | -1.43 | **12.00** | -1.98 | 0.27 | -2.48 | 2.87 | -2.52 | -1.63 |
| 6 | 115200000-115500000 | 1.60 | 0.96 | **11.45** | 0.27 | 5.39 | 1.25 | 0.20 | 5.13 | 1.79 | -0.61 |
| 7 | 3300000-3600000 | **8.38** | -0.93 | 0.97 | -1.39 | 4.81 | -1.51 | -1.04 | 1.94 | -0.54 | 2.12 |
| 7 | 14100000-14400000 | 1.73 | 0.79 | 6.41 | 0.65 | **7.77** | 2.15 | 5.74 | **15.54** | 2.50 | 0.77 |
| 7 | 25200000-25500000 | 1.30 | **9.96** | 0.49 | 0.06 | 0.32 | -0.57 | -2.35 | 1.81 | -0.47 | 5.36 |
| 7 | 26700000-27000000 | 3.49 | 1.56 | 4.74 | 3.99 | 0.74 | 1.06 | 6.92 | **10.39** | 2.37 | 1.59 |
| 7 | 27900000-28200000 | 0.99 | **13.62** | -0.66 | 0.94 | -1.09 | 1.13 | -2.52 | -1.42 | 1.30 | 0.57 |
| 7 | 32700000-33000000 | 0.21 | **9.89** | -0.55 | -0.66 | 0.54 | 1.02 | -0.47 | -0.80 | -1.02 | -2.13 |
| 7 | 51900000-52200000 | 2.27 | 2.70 | 3.42 | 0.70 | 3.44 | 0.87 | -1.81 | 1.66 | **12.54** | 1.53 |
| 7 | 57900000-58200000 | **9.93** | 7.24 | 3.79 | 8.38 | 5.95 | 3.07 | 10.63 | 4.83 | **11.92** | **12.32** |
| 7 | 64800000-65100000 | 1.45 | **10.56** | 1.88 | 0.19 | 0.96 | -0.05 | 2.39 | 0.34 | 2.08 | 0.22 |
| 7 | 65400000-65700000 | 0.77 | **9.72** | 1.62 | 2.19 | 1.14 | 2.02 | -0.63 | 0.89 | -0.03 | 6.49 |
| 7 | 67500000-67800000 | -0.46 | 3.08 | 0.80 | 0.69 | -0.84 | 0.30 | 0.57 | 3.99 | 0.97 | **9.51** |
| 7 | 68700000-69000000 | 1.59 | 6.79 | 1.28 | **8.66** | 6.06 | -0.32 | 3.07 | 1.70 | 1.38 | 5.38 |
| 7 | 72000000-72300000 | 0.28 | 7.66 | 2.15 | **11.23** | 2.56 | -0.63 | -1.56 | 0.77 | 1.04 | 1.64 |
| 7 | 78000000-78300000 | -2.51 | -0.29 | -2.14 | 0.71 | -2.12 | -1.59 | -1.65 | **11.50** | -2.65 | -1.61 |
| 7 | 79200000-79500000 | 0.03 | **10.70** | 0.73 | -1.85 | -1.62 | 0.36 | 1.11 | -1.74 | 0.35 | 0.63 |
| 7 | 81300000-81600000 | 2.04 | 4.03 | 0.53 | 2.60 | 2.06 | -0.08 | 0.78 | **9.78** | -0.94 | 0.77 |
| 7 | 81600000-81900000 | 0.98 | -0.46 | -1.06 | 8.38 | -0.31 | -1.33 | -2.10 | **11.06** | -1.66 | 0.79 |
| 7 | 82500000-82800000 | **9.12** | 7.83 | **16.44** | 5.86 | 4.38 | **9.33** | 5.53 | **9.54** | 4.50 | 4.42 |
| 7 | 89400000-89700000 | 4.00 | -0.22 | -0.15 | **13.22** | 1.87 | -0.71 | 0.78 | 1.85 | 2.12 | 0.15 |
| 7 | 99900000-100200000 | 1.95 | **11.20** | 3.43 | 1.51 | 3.35 | 2.89 | 7.57 | -0.66 | 5.43 | **11.26** |
| 8 | 4200000-4500000 | 7.01 | 3.98 | **11.77** | 7.53 | 2.55 | 5.60 | 3.27 | 6.06 | **11.24** | 6.17 |
| 8 | 8100000-8400000 | 3.19 | -0.68 | 1.66 | 0.57 | 4.24 | -0.78 | -0.06 | **9.85** | 2.01 | 2.42 |
| 8 | 12900000-13200000 | 2.54 | 4.00 | -1.68 | -0.70 | **9.59** | -0.07 | -0.60 | -1.23 | 0.72 | 0.00 |
| 8 | 13800000-14100000 | -1.04 | 3.11 | 4.58 | **8.99** | 0.50 | 0.43 | 6.92 | 4.94 | -0.70 | -1.04 |
| 8 | 16500000-16800000 | **8.53** | -0.66 | -0.47 | -1.17 | -0.18 | -1.97 | -0.87 | -1.40 | -1.12 | -0.94 |
| 8 | 21300000-21600000 | -1.48 | **8.65** | 0.59 | 0.96 | -0.85 | -0.26 | 0.61 | -0.72 | 2.22 | -0.11 |
| 8 | 24900000-25200000 | 1.20 | **10.62** | 1.91 | 3.04 | 3.38 | 0.81 | 2.91 | 1.24 | 0.74 | 0.39 |
| 8 | 32700000-33000000 | 0.28 | 1.82 | 0.20 | **9.98** | **7.48** | 2.53 | -1.28 | 5.17 | 1.10 | 5.60 |
| 8 | 40200000-40500000 | 3.58 | **12.10** | -0.46 | 3.52 | 1.91 | 1.58 | 2.34 | 0.31 | -0.04 | 0.09 |
| 8 | 41700000-42000000 | -1.08 | **9.86** | 0.52 | -0.28 | -2.53 | -2.39 | -0.79 | -0.52 | -2.05 | -2.51 |
| 8 | 42300000-42600000 | 4.49 | 4.21 | 4.13 | **12.66** | 2.47 | 1.80 | 6.20 | 3.98 | 0.91 | 0.97 |
| 8 | 51600000-51900000 | 0.04 | -1.98 | -1.42 | **11.56** | 3.89 | 0.08 | -0.14 | -0.76 | 2.07 | -0.26 |
| 8 | 73200000-73500000 | 3.63 | 2.74 | 0.32 | **9.32** | 2.60 | 1.93 | 7.13 | -0.59 | -0.26 | -0.12 |
| 8 | 74700000-75000000 | 0.68 | -1.11 | **7.87** | -0.27 | -2.65 | -1.13 | 6.46 | -0.29 | -0.62 | 2.72 |
| 8 | 87300000-87600000 | 2.34 | 1.89 | 3.79 | 2.50 | 0.81 | 2.35 | 1.83 | 1.36 | **11.78** | 3.57 |
| 8 | 87600000-87900000 | 0.94 | 2.31 | 1.56 | 3.60 | 2.93 | **11.70** | 8.34 | -0.74 | 1.64 | 1.49 |
| 8 | 90000000-90300000 | 0.77 | 0.67 | **12.85** | -1.98 | -1.06 | -1.09 | -2.89 | -1.96 | -0.63 | 1.00 |
| 9 | 6600000-6900000 | -0.75 | -2.59 | -0.95 | **11.55** | -0.42 | 2.48 | -2.52 | -1.48 | -0.05 | 0.28 |
| 9 | 6900000-7200000 | 1.52 | 0.33 | 0.05 | -0.75 | **7.81** | -0.84 | -0.52 | 6.56 | 0.82 | 4.65 |
| 9 | 27300000-27600000 | 3.85 | -0.63 | 1.63 | 5.12 | **7.52** | 1.17 | 0.98 | 5.62 | 5.51 | **14.05** |
| 9 | 35700000-36000000 | -0.24 | **9.55** | -0.70 | -1.68 | 3.15 | 1.37 | -1.58 | -1.78 | -1.84 | 0.07 |
| 9 | 47400000-47700000 | 1.76 | 2.48 | 3.04 | 1.23 | **8.15** | -0.73 | -0.36 | 7.91 | 4.01 | 2.54 |
| 9 | 52200000-52500000 | -0.71 | -1.88 | 6.52 | **11.22** | -0.20 | 1.36 | 3.09 | -0.49 | 3.87 | -1.15 |
| 9 | 59100000-59400000 | -1.57 | **10.78** | -0.17 | -2.44 | 1.22 | 1.04 | 2.98 | 2.34 | -0.27 | 0.17 |
| 9 | 72000000-72300000 | -1.71 | -2.82 | 0.04 | -1.83 | **9.76** | 0.58 | -0.66 | -1.04 | -2.82 | -0.03 |
| 9 | 73800000-74100000 | 3.00 | 2.28 | 5.83 | **11.29** | -0.77 | -1.78 | 1.74 | -0.11 | -1.64 | -1.86 |
| 9 | 74400000-74700000 | 2.20 | **11.49** | 3.34 | -0.38 | 6.35 | 2.52 | 2.04 | 2.84 | 0.92 | -0.06 |
| 9 | 77100000-77400000 | 4.63 | 1.00 | **11.79** | 0.48 | 1.64 | 0.07 | 0.49 | **10.96** | 6.49 | 7.59 |
| 9 | 77400000-77700000 | **13.04** | 4.74 | 3.42 | 0.67 | 1.62 | -0.42 | 0.66 | 3.60 | 1.12 | **9.37** |
| 9 | 77700000-78000000 | **13.30** | -0.51 | 3.82 | 1.71 | -0.35 | 1.60 | 4.06 | 4.55 | 3.81 | 4.60 |
| 9 | 78900000-79200000 | -2.15 | -2.23 | 2.24 | 2.19 | -0.38 | 1.16 | 0.31 | -1.14 | **7.96** | 2.30 |
| 9 | 85500000-85800000 | 2.70 | -1.31 | 2.03 | -0.72 | 0.64 | **13.61** | 7.55 | 0.07 | 1.06 | -0.37 |
| 9 | 87900000-88200000 | -0.71 | 3.33 | -0.22 | **8.81** | -1.01 | 0.58 | 5.06 | -2.38 | -0.73 | 6.18 |
| 9 | 88800000-89100000 | 4.25 | -0.42 | 3.98 | **20.53** | 0.87 | 3.25 | 4.83 | -0.41 | -0.21 | -0.12 |
| 9 | 93600000-93900000 | 0.12 | 2.52 | 3.12 | 5.56 | -0.40 | 1.54 | 0.15 | **10.64** | 1.31 | 1.27 |
| 9 | 94500000-94800000 | 0.22 | -2.34 | -0.86 | -0.50 | 0.41 | **10.58** | 4.52 | 4.28 | 0.70 | 2.72 |
| 10 | 6300000-6600000 | 0.31 | **13.29** | -0.70 | 0.05 | -0.88 | -0.91 | 0.95 | 0.09 | -1.18 | 0.16 |
| 10 | 6900000-7200000 | -1.88 | 6.25 | -0.48 | **9.39** | -1.77 | -2.69 | -3.63 | -3.72 | -2.87 | -3.92 |
| 10 | 8100000-8400000 | -1.64 | -1.23 | -0.13 | -1.74 | 7.00 | -1.01 | 5.47 | **10.36** | -3.06 | -0.83 |
| 10 | 9600000-9900000 | 0.72 | -1.12 | 3.28 | **9.42** | 3.88 | 1.10 | 0.39 | 3.25 | 0.32 | 4.53 |
| 10 | 10500000-10800000 | 5.77 | 5.00 | 3.70 | 6.28 | 3.88 | **8.52** | 9.88 | 5.15 | 4.20 | 5.12 |
| 10 | 18900000-19200000 | 0.91 | 0.93 | 4.36 | 1.03 | 1.10 | -0.23 | -0.03 | 7.99 | **16.24** | 6.98 |
| 10 | 26100000-26400000 | 0.35 | **13.50** | 2.25 | -2.39 | -1.05 | -2.57 | -1.44 | 2.04 | 0.88 | -2.59 |
| 10 | 28200000-28500000 | 1.92 | 0.79 | 2.60 | 0.46 | -1.26 | 0.47 | 1.17 | **10.71** | 3.02 | 1.89 |
| 10 | 28800000-29100000 | 1.12 | 3.33 | 4.13 | 4.39 | 1.84 | 0.31 | 4.73 | -0.20 | 2.13 | **9.35** |
| 10 | 29400000-29700000 | **9.80** | **12.93** | **14.44** | **10.74** | **12.18** | **10.47** | **18.24** | 6.72 | **30.11** | **33.24** |
| 10 | 30600000-30900000 | 4.79 | 4.43 | 4.35 | **15.98** | **7.63** | 3.70 | 6.29 | 4.90 | **9.37** | **15.78** |
| 10 | 30900000-31200000 | 1.94 | 2.43 | 4.65 | 0.54 | 0.85 | 1.75 | 0.65 | 1.52 | 4.43 | **12.35** |
| 10 | 32400000-32700000 | -1.10 | 2.18 | **7.90** | -1.52 | 1.63 | -0.31 | -1.22 | -0.49 | -0.61 | 4.27 |
| 10 | 33900000-34200000 | -0.18 | 3.60 | 0.83 | **15.36** | 0.05 | 2.90 | 0.38 | -0.33 | 1.00 | 1.27 |
| 10 | 35400000-35700000 | 2.48 | 4.59 | 5.43 | 5.86 | 3.92 | 2.24 | 5.04 | 5.62 | **7.91** | **17.39** |
| 10 | 35700000-36000000 | 3.07 | 1.68 | 1.73 | **8.97** | **12.99** | 2.88 | 2.11 | **15.81** | 4.85 | **17.20** |
| 10 | 36300000-36600000 | -0.73 | 1.23 | 1.12 | -1.47 | **9.95** | -1.01 | 2.48 | -0.06 | 0.48 | **16.69** |
| 10 | 41400000-41700000 | 3.87 | 0.58 | 0.35 | **9.09** | 6.87 | 4.43 | 0.76 | 2.87 | 4.72 | 5.04 |
| 10 | 42300000-42600000 | -1.49 | -1.40 | 4.54 | -2.41 | -0.61 | -4.05 | -2.54 | -5.07 | **9.26** | -2.83 |
| 10 | 42600000-42900000 | -1.60 | 2.44 | 5.91 | -2.76 | 1.18 | -2.46 | -1.82 | -3.21 | **8.54** | 2.87 |
| 10 | 52800000-53100000 | 5.02 | 4.66 | -0.66 | 4.78 | **7.51** | -0.23 | -1.11 | -1.72 | 2.23 | 1.07 |
| 10 | 55800000-56100000 | **8.47** | 0.22 | -1.53 | 0.32 | -0.74 | -1.75 | -1.68 | -1.71 | -1.91 | -0.48 |
| 10 | 61200000-61500000 | -1.42 | 2.92 | **9.28** | 1.02 | -0.26 | 1.25 | 0.65 | -2.77 | 1.47 | -1.36 |
| 10 | 68700000-69000000 | -0.38 | -0.01 | -1.09 | -0.67 | **8.80** | -0.14 | -0.51 | 6.10 | 2.38 | -0.65 |
| 10 | 73200000-73500000 | -0.82 | 2.61 | 0.49 | 0.47 | 0.01 | **9.03** | -2.05 | -0.39 | -0.66 | 0.80 |
| 10 | 81600000-81900000 | 2.83 | 2.51 | 4.57 | 3.50 | 5.57 | 2.58 | **12.08** | 2.84 | 2.23 | 2.32 |
| 11 | 3600000-3900000 | -0.57 | **10.45** | 0.80 | 1.57 | 4.83 | -1.21 | 5.67 | 0.45 | -1.36 | 3.99 |
| 11 | 14700000-15000000 | 4.50 | 1.39 | 5.85 | 0.98 | 2.40 | 0.59 | 0.11 | **10.17** | 1.12 | 1.09 |
| 11 | 18300000-18600000 | **8.00** | 4.74 | 5.76 | 4.89 | **16.43** | 7.80 | **17.63** | **20.80** | **10.63** | **19.90** |
| 11 | 20700000-21000000 | 3.38 | 1.29 | **7.29** | 2.45 | 1.81 | **16.32** | 1.92 | 0.31 | 0.21 | -1.13 |
| 11 | 26100000-26400000 | 2.15 | -0.69 | -0.20 | 1.87 | 1.05 | 7.21 | **11.23** | 1.28 | 3.29 | 0.05 |
| 11 | 26400000-26700000 | 1.37 | 2.80 | 0.65 | 3.70 | 1.45 | 0.53 | 1.88 | 5.01 | **7.98** | 5.47 |
| 11 | 27300000-27600000 | **9.02** | 3.17 | 5.07 | **11.26** | **10.35** | **9.67** | **16.86** | 5.54 | 7.35 | 8.90 |
| 11 | 30900000-31200000 | 2.71 | **8.68** | -0.05 | 1.07 | -0.92 | 0.40 | 0.21 | -0.34 | 1.92 | 6.93 |
| 11 | 31500000-31800000 | -0.48 | -2.12 | -1.84 | -1.75 | 1.47 | -2.68 | -0.26 | **12.40** | -1.63 | -0.21 |
| 11 | 31800000-32100000 | -1.91 | -0.20 | -2.30 | -0.21 | -1.09 | -1.92 | -2.28 | **9.66** | 2.70 | -0.80 |
| 11 | 42600000-42900000 | 1.23 | **10.48** | 1.83 | 4.36 | -1.92 | 0.80 | 0.65 | 1.56 | -0.20 | -2.12 |
| 11 | 46500000-46800000 | 0.72 | -0.28 | **9.85** | 0.94 | -1.49 | 0.30 | 2.62 | -2.06 | 3.40 | 1.57 |
| 11 | 49200000-49500000 | 0.83 | 1.99 | 0.28 | -0.70 | 0.68 | -0.22 | **14.57** | 4.97 | **8.78** | 1.07 |
| 11 | 49800000-50100000 | -1.06 | 0.82 | 0.24 | -0.13 | 2.43 | -0.05 | **11.73** | 0.76 | 0.40 | 1.48 |
| 11 | 55800000-56100000 | **9.43** | 5.67 | 4.30 | 3.97 | 0.97 | 4.99 | 0.78 | -2.69 | -0.43 | -0.91 |
| 12 | 23400000-23700000 | -0.52 | -0.53 | -0.44 | **10.37** | -0.24 | 1.30 | -1.18 | 0.03 | -0.36 | -1.37 |
| 12 | 25500000-25800000 | 0.51 | 0.58 | **9.09** | 5.08 | 3.67 | 0.02 | 0.57 | 3.04 | 5.48 | 3.47 |
| 12 | 32100000-32400000 | -1.55 | 0.16 | -0.95 | 4.43 | -0.56 | -0.70 | 1.19 | 1.86 | -1.17 | **9.60** |
| 12 | 33300000-33600000 | 0.87 | **10.07** | 1.52 | 3.52 | 1.93 | 1.57 | -1.15 | 0.95 | -1.16 | -0.78 |
| 12 | 34800000-35100000 | 0.18 | 1.95 | 2.72 | 0.18 | 1.77 | 0.86 | 2.71 | **9.70** | 0.14 | 1.22 |
| 12 | 39300000-39600000 | 2.55 | -0.94 | -1.90 | -0.48 | -1.26 | -1.66 | -3.03 | **10.84** | -2.35 | 4.65 |
| 12 | 42900000-43200000 | **11.28** | 5.61 | 0.70 | 1.05 | 0.89 | 1.62 | 7.57 | 4.68 | 1.45 | 1.42 |
| 12 | 51600000-51900000 | -0.69 | 4.03 | 0.07 | -3.48 | **8.04** | 0.80 | -0.72 | 4.16 | 1.36 | 2.26 |
| 12 | 56700000-57000000 | 3.78 | -0.03 | 1.32 | 2.27 | 1.00 | 0.77 | -0.78 | 4.69 | 3.19 | **14.01** |
| 12 | 60000000-60300000 | **16.47** | 0.80 | 0.91 | 2.62 | 1.39 | 6.42 | 1.00 | 3.63 | 3.84 | 3.25 |
| 12 | 68700000-69000000 | 0.10 | -0.18 | 1.20 | 0.21 | **8.56** | -1.24 | -0.60 | **15.74** | 2.75 | 5.19 |
| 12 | 72300000-72600000 | **8.11** | 6.33 | 2.84 | -0.05 | 0.69 | -1.92 | -0.74 | 0.26 | -0.68 | -1.89 |
| 13 | 1500000-1800000 | 0.63 | -2.56 | 0.19 | -0.19 | 0.45 | **8.40** | 4.27 | -0.91 | -1.30 | -2.53 |
| 13 | 12000000-12300000 | 4.70 | -0.90 | 4.50 | 5.56 | 4.96 | 4.39 | -1.51 | -2.15 | 0.69 | **14.53** |
| 13 | 17100000-17400000 | 0.89 | -0.89 | 2.09 | 2.44 | -0.18 | 0.26 | -0.18 | 4.50 | 2.74 | **15.60** |
| 13 | 20100000-20400000 | 2.34 | 0.94 | 2.01 | **9.01** | -1.31 | 0.71 | -0.30 | 5.68 | 1.13 | 0.56 |
| 13 | 22500000-22800000 | 0.50 | 0.38 | 4.17 | 1.21 | 0.11 | 1.50 | -0.13 | 1.08 | 0.83 | **12.59** |
| 13 | 27000000-27300000 | 6.17 | **10.56** | 3.09 | 5.16 | 4.27 | **8.04** | 1.73 | 7.42 | 6.96 | **17.78** |
| 13 | 32700000-33000000 | 1.25 | 0.65 | 2.64 | 5.03 | **7.94** | 3.69 | 0.88 | 4.20 | 4.22 | 2.98 |
| 13 | 36000000-36300000 | 1.94 | -0.10 | 1.25 | -1.43 | **8.62** | 0.46 | -1.94 | 1.25 | 3.09 | -1.01 |
| 13 | 36300000-36600000 | 0.55 | 0.37 | 3.58 | 1.54 | -1.24 | -0.91 | -1.34 | **11.77** | 1.36 | 0.58 |
| 13 | 42900000-43200000 | -0.95 | 1.88 | **7.19** | 5.22 | 5.53 | -1.24 | -0.36 | 2.20 | 0.80 | **10.61** |
| 13 | 43500000-43800000 | 0.96 | -0.46 | 6.39 | -1.30 | 0.29 | **10.01** | 3.58 | 1.29 | **9.21** | 1.97 |
| 13 | 45600000-45900000 | 2.05 | 1.31 | -0.85 | 0.57 | 2.41 | -0.26 | -2.56 | **9.60** | 3.78 | 3.76 |
| 13 | 47700000-48000000 | 4.07 | 1.45 | 3.54 | -0.21 | 1.14 | 1.68 | -1.43 | 5.50 | **7.71** | 6.27 |
| 13 | 48000000-48300000 | 6.38 | 0.76 | **9.36** | 3.77 | 4.57 | 1.79 | 1.18 | **13.62** | 6.87 | 2.70 |
| 13 | 48300000-48600000 | 4.09 | 7.08 | **11.17** | 4.18 | 4.10 | 3.55 | 8.29 | **19.95** | **10.09** | **13.33** |
| 13 | 48600000-48900000 | **11.15** | 6.73 | **15.82** | 6.50 | **8.62** | **12.12** | **11.18** | **19.17** | **11.41** | **23.95** |
| 13 | 48900000-49200000 | **15.94** | **11.54** | **16.19** | **13.34** | **12.28** | **12.52** | 8.88 | **31.92** | **28.62** | **35.07** |
| 13 | 51300000-51600000 | 0.18 | -1.86 | 2.00 | 0.27 | 2.46 | 1.76 | **23.53** | 5.41 | 3.65 | 5.35 |
| 13 | 53400000-53700000 | -0.45 | 0.31 | -0.45 | 0.97 | 1.45 | 0.60 | -1.44 | **12.09** | 1.11 | 0.27 |
| 13 | 58500000-58800000 | -0.13 | -1.60 | **11.53** | 2.46 | 0.82 | 1.19 | 1.35 | 0.51 | 2.33 | 3.25 |
| 13 | 59400000-59700000 | 1.40 | **8.94** | -0.52 | -0.94 | 1.93 | -1.51 | -2.62 | -1.36 | 0.30 | -2.08 |
| 13 | 60300000-60600000 | -0.28 | 3.69 | 4.98 | -1.55 | -0.40 | 2.32 | 4.54 | 0.73 | **8.60** | 0.99 |
| 13 | 62100000-62400000 | 2.02 | 4.11 | 3.38 | 2.93 | **8.23** | 2.94 | 0.47 | 2.73 | 1.65 | 0.72 |
| 13 | 62700000-63000000 | -0.37 | **8.82** | 5.63 | -1.42 | 1.93 | -0.56 | -0.90 | 3.29 | 0.99 | 3.75 |
| 13 | 63300000-63600000 | -0.73 | -1.74 | **11.49** | -1.00 | 1.57 | 1.41 | -2.15 | 0.64 | -1.82 | -1.40 |
| 13 | 64200000-64500000 | -1.76 | -2.73 | -2.61 | **9.98** | 1.12 | -2.13 | -3.40 | -0.23 | -2.55 | -1.09 |
| 13 | 79500000-79800000 | **7.83** | 6.70 | -1.04 | 7.58 | 3.32 | -0.23 | -0.07 | -1.03 | 3.96 | 0.24 |
| 13 | 79800000-80100000 | 1.02 | 3.57 | 5.34 | **10.23** | -0.69 | **11.49** | -2.24 | 0.31 | 1.40 | 1.16 |
| 13 | 81000000-81300000 | 0.88 | 1.00 | 1.23 | 2.94 | 6.62 | 1.79 | 2.41 | **12.78** | 3.58 | 1.17 |
| 14 | 3300000-3600000 | 2.35 | -2.21 | -1.63 | 0.49 | -0.69 | **13.40** | 2.58 | -0.96 | -0.08 | 6.61 |
| 14 | 10200000-10500000 | -1.11 | **9.30** | -0.10 | 5.26 | 0.33 | -0.10 | 3.30 | -1.97 | 4.32 | 2.47 |
| 14 | 14100000-14400000 | **10.19** | -0.88 | 1.30 | 4.91 | 0.30 | 0.48 | 4.06 | 2.22 | 1.13 | 6.29 |
| 14 | 18000000-18300000 | 3.79 | 1.82 | 2.89 | -1.02 | **12.37** | 0.00 | -3.27 | 2.15 | 1.08 | 0.99 |
| 14 | 23700000-24000000 | 0.40 | **9.69** | 1.96 | -1.60 | 4.24 | 0.68 | 0.00 | -2.69 | -0.15 | 3.02 |
| 14 | 32400000-32700000 | **8.45** | -0.44 | -0.40 | -1.19 | -2.16 | -0.53 | -2.61 | -1.51 | 0.03 | 4.83 |
| 14 | 38100000-38400000 | 0.19 | 5.73 | 1.49 | -0.69 | **13.19** | -0.22 | -0.54 | **9.65** | 2.62 | 5.22 |
| 14 | 39300000-39600000 | 0.97 | 0.17 | 3.20 | 5.90 | 1.87 | **10.26** | 2.58 | -0.35 | 1.18 | **11.34** |
| 14 | 48900000-49200000 | 7.34 | 0.91 | 0.28 | 4.01 | 2.79 | -0.61 | 1.14 | **9.28** | 3.53 | 6.51 |
| 14 | 53400000-53700000 | -1.26 | -3.55 | -0.11 | -2.04 | -0.71 | -2.67 | 1.13 | -1.88 | -2.63 | **9.86** |
| 14 | 56700000-57000000 | 0.61 | -1.10 | 0.01 | 2.74 | 2.45 | 3.25 | **14.15** | -1.61 | 0.12 | **11.71** |
| 14 | 59100000-59400000 | 0.24 | 3.61 | 4.03 | 2.83 | 0.98 | 2.30 | -0.59 | **14.87** | 1.30 | 2.96 |
| 14 | 60000000-60300000 | 2.50 | 2.21 | 1.37 | -1.75 | **13.10** | 1.45 | 1.76 | 1.18 | 2.05 | 1.87 |
| 15 | 900000-1200000 | 4.75 | **13.57** | **11.25** | **14.56** | 4.47 | 7.34 | **14.92** | **11.33** | 5.74 | **15.67** |
| 15 | 3000000-3300000 | **8.85** | 2.39 | 3.36 | 1.05 | 0.77 | 2.34 | 6.14 | 3.09 | 1.72 | 4.20 |
| 15 | 3300000-3600000 | **9.03** | 7.65 | **11.10** | **13.55** | **9.72** | **12.85** | 9.58 | **21.96** | **22.17** | **26.52** |
| 15 | 3600000-3900000 | **8.32** | 6.62 | **12.09** | 8.13 | **8.85** | **8.00** | 8.14 | **18.00** | **32.89** | **17.87** |
| 15 | 4800000-5100000 | -0.62 | 3.44 | **8.35** | 4.26 | 1.65 | 2.25 | -0.84 | 1.45 | -0.62 | 1.08 |
| 15 | 17100000-17400000 | -1.11 | 2.40 | **7.57** | 1.52 | -0.34 | 5.87 | -0.12 | -1.14 | 1.63 | -2.60 |
| 15 | 27300000-27600000 | 4.89 | 4.71 | 3.89 | 6.21 | 4.35 | **8.69** | 10.42 | 0.99 | 1.80 | 7.36 |
| 15 | 28500000-28800000 | -1.55 | 0.02 | 0.14 | -0.96 | 5.36 | 0.16 | -2.59 | -0.51 | **10.41** | 0.60 |
| 15 | 33000000-33300000 | 6.27 | 0.56 | 4.11 | 6.51 | 1.28 | 2.11 | 2.19 | 0.30 | **9.07** | **12.15** |
| 15 | 38400000-38700000 | 1.72 | 3.13 | 1.86 | **16.87** | 2.28 | -0.21 | -1.72 | -1.25 | 6.51 | 4.59 |
| 15 | 38700000-39000000 | **9.09** | 1.23 | 0.55 | 2.75 | 2.58 | 6.27 | -1.38 | -2.98 | 0.02 | -0.90 |
| 15 | 42000000-42300000 | 0.54 | 0.57 | -0.26 | **12.55** | 2.66 | 6.79 | 1.01 | 1.12 | 6.35 | 5.35 |
| 15 | 49800000-50100000 | -1.83 | -2.12 | -0.21 | -1.49 | -2.19 | 1.01 | 4.17 | **19.32** | 1.54 | 0.35 |
| 15 | 54300000-54600000 | 0.87 | 1.11 | 2.74 | 2.45 | 2.97 | 1.62 | 1.68 | **20.18** | 4.79 | 2.50 |
| 15 | 54600000-54900000 | 5.82 | 2.61 | 0.40 | 3.62 | 1.88 | -0.63 | 3.00 | 3.74 | **8.61** | 1.21 |
| 15 | 55200000-55500000 | -2.19 | 3.01 | 0.72 | **9.34** | -2.31 | 4.06 | -2.29 | -0.33 | -0.68 | -0.44 |
| 15 | 66000000-66300000 | -1.94 | -2.63 | 0.08 | -0.18 | -1.99 | 1.12 | -3.11 | **12.11** | -2.18 | -2.13 |
| 15 | 66300000-66600000 | 3.41 | -1.15 | -0.28 | -0.76 | 3.00 | **8.10** | -0.06 | 1.52 | 2.42 | 4.28 |
| 15 | 72300000-72600000 | -0.19 | 2.65 | 1.48 | 7.51 | -0.10 | 5.04 | 1.76 | 0.76 | **11.77** | 3.03 |
| 15 | 78000000-78300000 | **14.11** | -3.00 | 0.46 | 3.46 | 2.37 | -1.08 | 2.36 | -2.71 | 0.96 | 0.29 |
| 15 | 79500000-79800000 | 4.09 | 1.66 | **7.93** | -0.53 | 6.66 | 5.57 | 1.36 | 4.26 | 2.75 | 0.49 |
| 16 | 4800000-5100000 | 3.00 | 0.01 | -0.33 | 2.55 | 0.29 | 3.98 | **10.98** | 6.44 | 2.67 | 1.65 |
| 16 | 5100000-5400000 | 2.74 | 0.51 | 1.16 | 5.36 | 1.65 | 3.02 | 9.08 | 2.79 | **7.79** | 2.20 |
| 16 | 8400000-8700000 | 4.03 | **9.16** | **9.05** | 4.40 | 2.34 | -0.45 | 4.45 | 1.31 | 1.02 | -0.44 |
| 16 | 10500000-10800000 | 0.70 | 0.16 | 2.78 | 5.17 | 4.37 | 0.55 | 0.97 | 4.01 | **8.38** | 0.81 |
| 16 | 16800000-17100000 | 0.95 | **17.75** | 0.17 | 1.30 | 4.03 | 1.23 | 4.33 | 3.68 | 4.86 | 0.86 |
| 16 | 25200000-25500000 | **11.78** | 0.90 | 0.30 | 0.24 | 1.93 | 3.30 | 0.88 | 6.36 | 4.46 | 7.71 |
| 16 | 33600000-33900000 | -0.87 | 2.30 | 1.37 | 0.68 | -0.94 | -2.06 | -2.21 | **14.02** | -0.42 | -0.46 |
| 16 | 34500000-34800000 | **16.42** | 4.36 | 5.38 | 5.15 | 3.99 | 2.35 | 3.77 | 4.43 | **13.61** | 6.26 |
| 16 | 37500000-37800000 | 3.86 | -0.05 | -0.43 | 0.21 | **8.93** | -0.92 | 0.51 | 1.04 | 4.52 | -0.48 |
| 16 | 40500000-40800000 | -0.50 | -0.99 | 1.06 | -0.34 | 2.16 | **10.30** | 0.91 | -0.82 | 2.74 | 8.68 |
| 16 | 51900000-52200000 | -2.46 | -2.04 | -2.56 | 0.46 | -0.55 | -1.91 | 1.03 | 0.04 | **9.53** | -0.23 |
| 16 | 63900000-64200000 | -0.83 | 4.33 | 2.45 | **13.74** | 3.19 | 2.05 | -2.25 | 1.68 | -0.52 | 3.59 |
| 16 | 70500000-70800000 | -0.51 | -0.26 | 1.69 | -2.90 | 2.18 | -3.29 | -0.41 | **9.72** | 0.06 | -3.18 |
| 16 | 71400000-71700000 | 4.35 | -0.22 | 1.07 | 0.88 | 1.77 | 6.32 | -0.48 | 1.23 | **14.07** | 3.93 |
| 17 | 900000-1200000 | 1.92 | 4.14 | 1.55 | 2.84 | 0.97 | -0.17 | **12.01** | 5.20 | -1.04 | -1.08 |
| 17 | 15900000-16200000 | 1.06 | -2.29 | 0.30 | 0.00 | **8.89** | 0.03 | -1.73 | 0.58 | -0.60 | **9.21** |
| 17 | 21900000-22200000 | 5.01 | 2.43 | 0.19 | 1.87 | -0.44 | **8.52** | -0.30 | 0.48 | 1.66 | 3.80 |
| 17 | 29100000-29400000 | 0.46 | 1.12 | 0.94 | 1.49 | 4.67 | -0.47 | 3.75 | 5.57 | **8.21** | 8.23 |
| 17 | 29400000-29700000 | 4.85 | -1.21 | 1.99 | 4.03 | 1.59 | -0.43 | -1.58 | **13.67** | 1.57 | 1.28 |
| 17 | 31500000-31800000 | **10.25** | 0.00 | 3.42 | 0.63 | 1.74 | 1.32 | 7.72 | 1.58 | -0.83 | -1.94 |
| 17 | 51300000-51600000 | 0.42 | 1.95 | 0.39 | -1.81 | -2.53 | **8.47** | -2.21 | 0.14 | 1.10 | 5.10 |
| 17 | 53100000-53400000 | 4.30 | 2.63 | **9.05** | 3.11 | 2.23 | 0.42 | 0.41 | 8.69 | 0.96 | 5.15 |
| 17 | 53700000-54000000 | 0.31 | 0.42 | -2.42 | -1.61 | -0.80 | **8.01** | 0.56 | 1.62 | -0.32 | 1.53 |
| 17 | 54600000-54900000 | **7.97** | -0.70 | -0.24 | 2.13 | 2.18 | **11.86** | 3.26 | 3.74 | 5.13 | 0.72 |
| 17 | 62100000-62400000 | -0.32 | **8.63** | -0.29 | -1.13 | -0.70 | -0.19 | -0.75 | 4.06 | -2.54 | -1.68 |
| 17 | 62700000-63000000 | 2.40 | 0.31 | **9.01** | 0.45 | 2.98 | 1.41 | 1.84 | -0.60 | 0.70 | 1.38 |
| 17 | 69600000-69900000 | 0.28 | 4.85 | 0.59 | 3.33 | 0.87 | 0.23 | -0.53 | 5.09 | **8.06** | -0.53 |
| 18 | 6000000-6300000 | -1.43 | 4.54 | -1.07 | -2.60 | -1.82 | 0.55 | -2.75 | 5.57 | -0.57 | **12.19** |
| 18 | 17100000-17400000 | -1.56 | -0.27 | 0.30 | **9.20** | 2.56 | -2.78 | 2.88 | 1.69 | 0.57 | -0.49 |
| 18 | 19200000-19500000 | 0.30 | 2.27 | 0.79 | **9.16** | 1.70 | -1.10 | 0.42 | 0.15 | 4.81 | 3.88 |
| 18 | 22800000-23100000 | 4.72 | 1.83 | 4.42 | 5.36 | 0.42 | 0.89 | -1.59 | 4.58 | 0.65 | **10.17** |
| 18 | 24900000-25200000 | 4.66 | 4.23 | 1.20 | 4.64 | 2.00 | 3.36 | **11.32** | 7.21 | 2.74 | **10.15** |
| 18 | 25800000-26100000 | 4.27 | 1.55 | 6.09 | 0.41 | 5.00 | 1.22 | 6.19 | 1.76 | 0.54 | **9.10** |
| 18 | 29400000-29700000 | 1.28 | 1.78 | 1.94 | 0.14 | 3.28 | 1.45 | **14.42** | 5.34 | 2.63 | 5.41 |
| 18 | 30900000-31200000 | 6.84 | -0.54 | **7.83** | -0.21 | 0.47 | 7.43 | 6.78 | -1.41 | 0.00 | 3.51 |
| 18 | 40800000-41100000 | 4.02 | 2.02 | 2.46 | -0.01 | **9.22** | 4.08 | 2.83 | 4.93 | 0.25 | 0.31 |
| 18 | 44400000-44700000 | **8.04** | 0.46 | 3.20 | -0.41 | 3.91 | **9.14** | 2.15 | 1.02 | 0.50 | 4.86 |
| 18 | 46800000-47100000 | 0.51 | 4.99 | -0.64 | 3.21 | 0.61 | -0.09 | 0.16 | **9.47** | 1.86 | -1.14 |
| 18 | 48300000-48600000 | **8.25** | 1.26 | 0.44 | 1.46 | 1.12 | 1.47 | 2.90 | -1.01 | 3.09 | -1.16 |
| 18 | 51600000-51900000 | -1.43 | -2.17 | -1.81 | -2.43 | -1.35 | **9.75** | 1.24 | -0.54 | -1.09 | 5.87 |
| 18 | 64200000-64500000 | 2.02 | 2.79 | 0.48 | 0.50 | **8.00** | 1.17 | 4.45 | 3.42 | 3.53 | 5.24 |
| 19 | 4200000-4500000 | 2.69 | 4.91 | 4.51 | 1.55 | 2.22 | 1.60 | **11.06** | 2.77 | 3.20 | 8.37 |
| 19 | 12000000-12300000 | 1.81 | -0.31 | -1.98 | 2.51 | **8.56** | -1.09 | 4.32 | 2.58 | -0.83 | -1.14 |
| 19 | 13800000-14100000 | 0.51 | 3.03 | **8.77** | 0.82 | -0.99 | -0.90 | -2.00 | -1.55 | -1.27 | 2.84 |
| 19 | 19800000-20100000 | -0.33 | 0.27 | -0.56 | **11.33** | 3.28 | -1.54 | 1.70 | 2.56 | 1.56 | 2.90 |
| 19 | 24300000-24600000 | 2.36 | 3.11 | -0.21 | 5.37 | 2.42 | **8.12** | 0.63 | 4.18 | 0.50 | 1.43 |
| 19 | 27000000-27300000 | 6.34 | -1.27 | **7.87** | -0.83 | -1.02 | 1.70 | 0.30 | 1.19 | -0.61 | 0.16 |
| 19 | 27300000-27600000 | -0.49 | -2.43 | **8.76** | -0.44 | 6.35 | 0.93 | 1.50 | 2.20 | 0.38 | -1.07 |
| 19 | 27600000-27900000 | 0.35 | 7.63 | 3.60 | -0.37 | **7.61** | -0.88 | 0.53 | -0.61 | 2.64 | **9.72** |
| 19 | 28800000-29100000 | 0.65 | **13.36** | 6.51 | 3.05 | 1.83 | -0.01 | -0.64 | 1.79 | 3.68 | **11.66** |
| 19 | 33600000-33900000 | -2.69 | 0.58 | -0.39 | 0.61 | 0.66 | 0.82 | **11.22** | -0.14 | -0.19 | 0.14 |
| 19 | 41400000-41700000 | -1.06 | 2.66 | 1.84 | -1.22 | -0.43 | **9.59** | 1.09 | -0.76 | -1.58 | -1.07 |
| 19 | 43200000-43500000 | **9.41** | 2.17 | -0.99 | -1.79 | 1.16 | -0.24 | -3.21 | -0.77 | -2.00 | 1.14 |
| 19 | 46800000-47100000 | **8.93** | 8.32 | 2.48 | 5.94 | -1.25 | -0.52 | -1.10 | 1.62 | 4.63 | 4.85 |
| 19 | 51300000-51600000 | -2.91 | -2.08 | -2.58 | -1.58 | 1.86 | **13.65** | -1.60 | -2.02 | -2.25 | 0.90 |
| 19 | 53700000-54000000 | 1.62 | -1.43 | 0.78 | **12.35** | -1.22 | -1.05 | -1.24 | -0.92 | 1.02 | 4.13 |
| 19 | 58800000-59100000 | **10.23** | 2.75 | 6.36 | 2.72 | 1.68 | 1.94 | 4.91 | 0.30 | 2.73 | 6.02 |
| 20 | 1200000-1500000 | **10.93** | -2.75 | -0.67 | -0.72 | -1.85 | 0.24 | 2.59 | -1.46 | -2.02 | -0.69 |
| 20 | 2400000-2700000 | 2.18 | 1.76 | -2.41 | **8.89** | -1.13 | -1.28 | -3.31 | -1.83 | 0.04 | 1.22 |
| 20 | 9000000-9300000 | 1.48 | 7.93 | 1.49 | 1.49 | 0.65 | 5.92 | -1.29 | -0.62 | **9.85** | 2.41 |
| 20 | 14400000-14700000 | 3.73 | **9.17** | -0.74 | 1.05 | 0.06 | -1.05 | -2.53 | 1.19 | -1.69 | 1.93 |
| 20 | 20100000-20400000 | 0.54 | 0.24 | **9.86** | 1.02 | 2.96 | 0.40 | -1.86 | 2.60 | -1.45 | -1.35 |
| 20 | 48900000-49200000 | 2.85 | 3.88 | 2.16 | 2.96 | 0.31 | **9.08** | -0.85 | -0.06 | 3.27 | 4.46 |
| 20 | 50700000-51000000 | -0.33 | 1.21 | 5.68 | 1.88 | 1.23 | -0.58 | 2.44 | -2.11 | -0.66 | **9.45** |
| 21 | 300000-600000 | 1.77 | -1.04 | 2.39 | 0.76 | -0.07 | **10.64** | 5.29 | 3.21 | 4.39 | 0.57 |
| 21 | 1800000-2100000 | 1.50 | 3.87 | **13.42** | 4.96 | 1.91 | 0.37 | 0.20 | 2.14 | 1.74 | 0.99 |
| 21 | 2100000-2400000 | 4.22 | -0.12 | **11.97** | 0.37 | 1.84 | -0.93 | 1.04 | 5.81 | 4.55 | 0.91 |
| 21 | 3900000-4200000 | 1.35 | 4.94 | -0.31 | **11.99** | -0.13 | 0.87 | 1.94 | 0.56 | 3.79 | 1.00 |
| 21 | 7800000-8100000 | -0.33 | 0.80 | 1.88 | **10.12** | -1.40 | -1.04 | 1.06 | -1.05 | 3.09 | 4.58 |
| 21 | 9000000-9300000 | 2.97 | -2.39 | 0.65 | 1.90 | 0.27 | -0.15 | **14.44** | -3.68 | -2.62 | 0.00 |
| 21 | 10200000-10500000 | -0.57 | 0.34 | 1.69 | 0.75 | 0.59 | 0.42 | **11.28** | 5.71 | 1.00 | 3.32 |
| 21 | 10500000-10800000 | 2.98 | -0.89 | 3.12 | 1.19 | 1.02 | 1.06 | 0.45 | **9.93** | -0.95 | -1.91 |
| 21 | 15900000-16200000 | -0.51 | 4.66 | 2.34 | 3.36 | -0.75 | 0.66 | 5.91 | **11.67** | 0.57 | -0.61 |
| 21 | 24300000-24600000 | -1.19 | 1.45 | -0.60 | **8.61** | -0.05 | 0.14 | -2.28 | 3.10 | 1.55 | -0.39 |
| 21 | 24900000-25200000 | 0.69 | **12.55** | 1.70 | 0.99 | 1.48 | **9.62** | 0.82 | 0.96 | 7.34 | 6.55 |
| 21 | 27900000-28200000 | 0.11 | -2.46 | -1.53 | 0.73 | -0.25 | 1.10 | 6.14 | **12.31** | -1.38 | -1.36 |
| 21 | 36600000-36900000 | 2.62 | 1.34 | -0.10 | -2.46 | 1.14 | 1.99 | -1.61 | 3.26 | 0.01 | **9.53** |
| 21 | 39900000-40200000 | 2.50 | 1.93 | **7.32** | 6.84 | 2.66 | 3.02 | 0.52 | 3.32 | 3.27 | 3.04 |
| 21 | 44400000-44700000 | 0.60 | 0.08 | 2.62 | 7.19 | 1.61 | **10.86** | -0.71 | -0.47 | **7.82** | 1.06 |
| 21 | 45300000-45600000 | 0.09 | 0.63 | **9.05** | -1.46 | 1.00 | -0.53 | -2.67 | 7.38 | 1.57 | 4.29 |
| 21 | 45900000-46200000 | 2.39 | **11.63** | 0.46 | 1.85 | 0.64 | -1.92 | 2.84 | -0.22 | 3.49 | -1.27 |
| 22 | 300000-600000 | 0.77 | 0.82 | -0.43 | 3.83 | 0.98 | 7.43 | 5.69 | **12.48** | 2.31 | -1.30 |
| 22 | 1200000-1500000 | 1.76 | 2.04 | 0.24 | 1.80 | 1.30 | **10.32** | 2.80 | -0.43 | 6.90 | -0.26 |
| 22 | 3000000-3300000 | -0.77 | -2.32 | 0.28 | -1.38 | 2.17 | 1.02 | 5.00 | -0.03 | **7.65** | -2.06 |
| 22 | 8100000-8400000 | 3.37 | 0.73 | 1.31 | 4.76 | 5.54 | 1.75 | -0.43 | **9.85** | 0.59 | 3.81 |
| 22 | 8700000-9000000 | -0.18 | 0.03 | 0.48 | 1.85 | **8.58** | -0.86 | -0.03 | 8.21 | 3.58 | 0.74 |
| 22 | 16500000-16800000 | 7.42 | -0.64 | -0.68 | 0.02 | 0.97 | -0.62 | 3.35 | 0.47 | 2.84 | **9.46** |
| 22 | 16800000-17100000 | 5.08 | **9.06** | -0.32 | -0.79 | -0.89 | 0.94 | -1.04 | -1.69 | -0.13 | -2.28 |
| 22 | 37800000-38100000 | 1.77 | **9.67** | -1.21 | 2.77 | 2.39 | 2.65 | -0.45 | 2.34 | -1.20 | -0.51 |
| 22 | 38100000-38400000 | 4.10 | 2.30 | 0.41 | 1.46 | 0.72 | 2.96 | -1.31 | 3.14 | 0.77 | **10.65** |
| 22 | 44400000-44700000 | 2.78 | 2.61 | 6.09 | **9.15** | 1.30 | 7.63 | 4.49 | 1.18 | 2.83 | 7.49 |
| 22 | 46500000-46800000 | 0.37 | -1.07 | 0.97 | -2.27 | -0.66 | **8.68** | -1.47 | -1.15 | 3.97 | -1.12 |
| 23 | 2100000-2400000 | **8.51** | -2.10 | 1.37 | 1.11 | 0.21 | 1.86 | 5.69 | 0.22 | 1.50 | -0.34 |
| 23 | 15600000-15900000 | 0.57 | -1.19 | 3.19 | 0.65 | 0.99 | **10.56** | 1.24 | 1.50 | 1.66 | -1.39 |
| 23 | 16500000-16800000 | 1.71 | -2.45 | -0.56 | 5.56 | -0.77 | **15.97** | 0.22 | -0.74 | -0.70 | 3.18 |
| 23 | 20100000-20400000 | 0.88 | -0.01 | **10.13** | -0.72 | -1.64 | -0.11 | 1.27 | -0.77 | 3.86 | 3.74 |
| 23 | 23100000-23400000 | 0.01 | -1.80 | 1.35 | -0.72 | -0.26 | 0.48 | 10.03 | **17.27** | -0.65 | -1.58 |
| 23 | 24300000-24600000 | 4.62 | 1.81 | 1.06 | 1.89 | **16.51** | **8.06** | 2.73 | 1.03 | 1.90 | 7.40 |
| 23 | 25200000-25500000 | **7.84** | 0.11 | 3.97 | 2.74 | 1.73 | 1.24 | 1.61 | 5.89 | 3.12 | 3.50 |
| 23 | 27000000-27300000 | -1.35 | 1.23 | 0.09 | 1.48 | **7.98** | 4.28 | 1.99 | 0.78 | 1.87 | **9.30** |
| 23 | 29400000-29700000 | 1.04 | 0.25 | 3.55 | -0.37 | 1.97 | **9.66** | 5.28 | -1.55 | 2.51 | 1.51 |
| 23 | 30600000-30900000 | -0.89 | 2.27 | **7.07** | 3.46 | 0.39 | 1.34 | 7.05 | -2.49 | 1.52 | -0.59 |
| 23 | 33600000-33900000 | 5.66 | 2.62 | 1.31 | 1.23 | 2.81 | **8.80** | 8.74 | -0.16 | 3.15 | 2.68 |
| 23 | 36300000-36600000 | 3.92 | -0.12 | 0.65 | -0.73 | -1.44 | **10.87** | 1.88 | -0.82 | 2.06 | -0.68 |
| 23 | 36900000-37200000 | -0.10 | 3.70 | 1.50 | 0.22 | 2.23 | 0.57 | 4.16 | 1.87 | **8.31** | 7.24 |
| 23 | 47400000-47700000 | 1.78 | 0.11 | **7.61** | 3.38 | 0.64 | 4.52 | 2.88 | 4.64 | 3.21 | **9.89** |
| 23 | 60600000-60900000 | -1.22 | -2.58 | -1.58 | -3.12 | 1.77 | 6.16 | -3.40 | -2.75 | **9.55** | 1.15 |
| 23 | 62100000-62400000 | 0.61 | **9.93** | **10.22** | -1.35 | 6.89 | -0.12 | -2.31 | 0.17 | 2.19 | 1.47 |
| 24 | 2400000-2700000 | 0.00 | -0.61 | -0.54 | 0.24 | -0.81 | **8.63** | 1.53 | 2.38 | 1.94 | -1.18 |
| 24 | 2700000-3000000 | **12.33** | 0.24 | 2.61 | -0.56 | 0.82 | 3.38 | 2.71 | 4.68 | 2.88 | -1.16 |
| 24 | 9900000-10200000 | 1.45 | **12.91** | -0.33 | 0.36 | 5.57 | 0.38 | -1.41 | -0.38 | 2.35 | 0.43 |
| 24 | 13500000-13800000 | 1.14 | -1.40 | -0.44 | **10.18** | 0.47 | -1.10 | -0.36 | -0.60 | 2.99 | -0.76 |
| 24 | 26100000-26400000 | -1.07 | 7.68 | 0.50 | 3.69 | 4.16 | -0.36 | 2.64 | **11.28** | 0.89 | 0.51 |
| 24 | 26700000-27000000 | **16.57** | 0.03 | 3.61 | -0.53 | -0.65 | -0.10 | -3.42 | -2.75 | -2.29 | -3.61 |
| 24 | 37500000-37800000 | 5.81 | 1.59 | 2.91 | 0.04 | 1.95 | **15.54** | 0.43 | 1.07 | 4.31 | 3.16 |
| 24 | 39000000-39300000 | 1.16 | -1.93 | 0.99 | 0.50 | -2.12 | -3.17 | -1.14 | 1.04 | -2.23 | **10.69** |
| 24 | 39300000-39600000 | **9.52** | -3.32 | -1.93 | -0.14 | 2.66 | -0.17 | 5.20 | -3.86 | 0.64 | -3.46 |
| 25 | 7200000-7500000 | 4.80 | 4.26 | **8.38** | **15.20** | 3.03 | 7.77 | **14.88** | 1.37 | 3.96 | 8.59 |
| 25 | 7500000-7800000 | 6.70 | **10.02** | **8.77** | 7.65 | 4.91 | 4.36 | **29.66** | 8.41 | 7.47 | **13.39** |
| 25 | 12000000-12300000 | -0.74 | 1.79 | **8.87** | 0.68 | -0.67 | -2.42 | -1.56 | 1.66 | 1.75 | -0.61 |
| 25 | 21300000-21600000 | -4.15 | 0.09 | **8.33** | -2.14 | -0.79 | -0.61 | -1.35 | 2.74 | -2.43 | 0.71 |
| 25 | 28200000-28500000 | -2.58 | **9.92** | -1.02 | -0.04 | -2.89 | -1.21 | 0.25 | -2.48 | -0.51 | 1.75 |
| 25 | 28800000-29100000 | 2.05 | 2.39 | 1.68 | 4.18 | 1.24 | 4.68 | **11.90** | 8.98 | 3.24 | 0.54 |
| 25 | 29100000-29400000 | 0.54 | -0.61 | 1.12 | 3.48 | 2.33 | **8.61** | 1.62 | -0.05 | 0.29 | 2.16 |
| 25 | 29400000-29700000 | 0.88 | 0.86 | -0.08 | 0.30 | 0.97 | -0.40 | 2.18 | **9.83** | 2.78 | -0.90 |
| 25 | 30600000-30900000 | 0.51 | 1.14 | -1.40 | -1.06 | -1.89 | -0.56 | 1.15 | 0.61 | 3.78 | **10.84** |
| 25 | 36000000-36300000 | 1.41 | 1.08 | 2.14 | 5.79 | -0.75 | **9.62** | -2.60 | -0.61 | 2.79 | -2.11 |
| 25 | 40800000-41100000 | -0.21 | 2.49 | 3.07 | -0.62 | 1.38 | 6.00 | 3.00 | -0.44 | **7.60** | 0.92 |
| 26 | 0-300000 | -0.27 | 0.45 | **11.10** | -0.27 | 2.72 | 0.18 | 1.56 | **10.13** | 0.21 | -0.28 |
| 26 | 600000-900000 | -1.97 | -1.73 | 2.19 | -2.51 | -1.05 | -2.80 | -2.49 | **12.97** | -2.05 | -0.26 |
| 26 | 6600000-6900000 | 2.78 | **9.54** | 0.30 | 0.69 | 1.38 | -0.69 | 1.05 | 2.32 | 0.44 | 5.11 |
| 26 | 8700000-9000000 | 2.11 | 0.68 | 6.38 | 1.15 | **7.53** | -0.69 | 1.00 | 3.67 | 0.88 | -0.10 |
| 26 | 18300000-18600000 | **9.58** | 1.38 | 1.46 | -0.15 | 0.14 | -1.35 | -1.04 | -0.90 | -0.95 | 0.62 |
| 26 | 24900000-25200000 | 3.29 | 0.74 | -2.06 | -1.91 | 0.51 | -2.08 | 1.17 | -1.54 | **7.79** | 0.85 |
| 26 | 25800000-26100000 | 3.86 | -0.91 | 2.71 | 1.37 | 1.63 | 2.71 | 1.39 | -0.35 | **8.27** | -1.16 |
| 26 | 43200000-43500000 | -0.30 | 3.61 | 2.69 | -0.08 | **8.33** | 1.82 | -0.50 | 1.92 | 5.98 | -0.07 |
