# Supplementary material for: Genome-wide analysis reveals population structure and selection in Chinese indigenous sheep breeds
Source: BMC Genomics. 2015 Mar 17;16(1):194. doi: 10.1186/s12864-015-1384-9 (PMC4404018; doi:10.1186/s12864-015-1384-9)
Supplement: Additional file 10: Table S5. — Images of 10 Chinese indigenous sheep breeds. [file 12864_2015_1384_MOESM10_ESM.docx]

**Table S5. Images of 10 Chinese indigenous sheep breeds**

| Breed name | Pictures of Ewe |
| --- | --- |
| UJI | 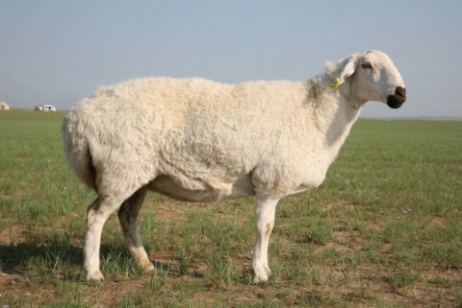 |
| HUS | 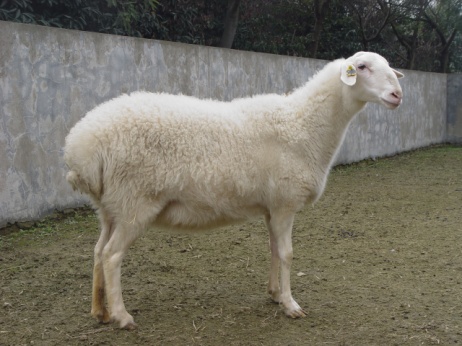 |
| TON | 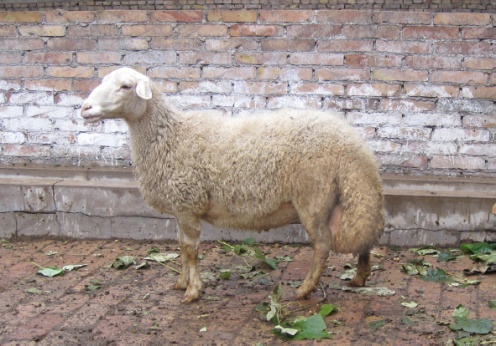 |
| LTH | 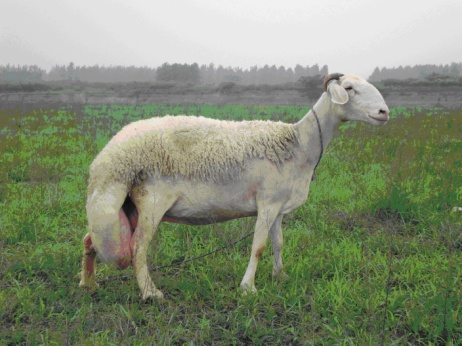 |
| LOP | 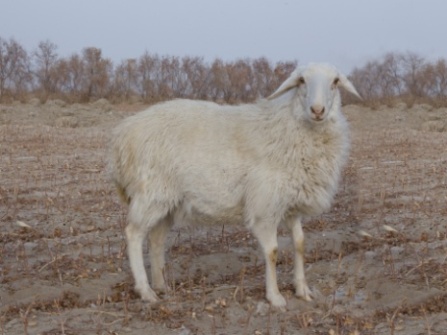 |
| KAZ | 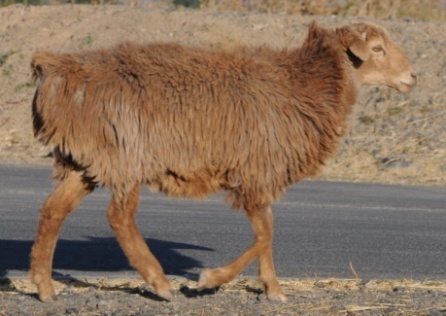 |
| DUL | 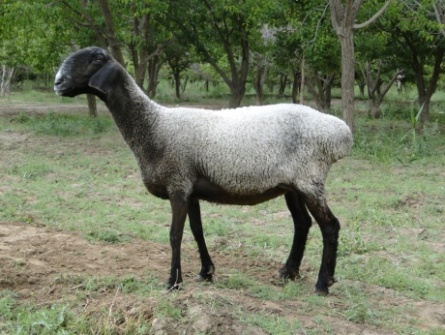 |
| DIQ | 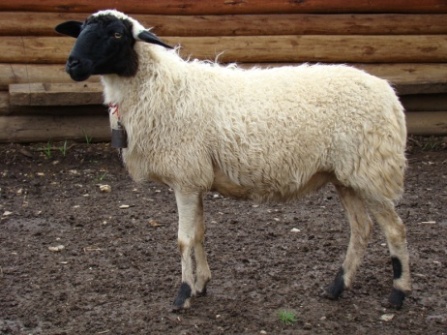 |
| TIBV | 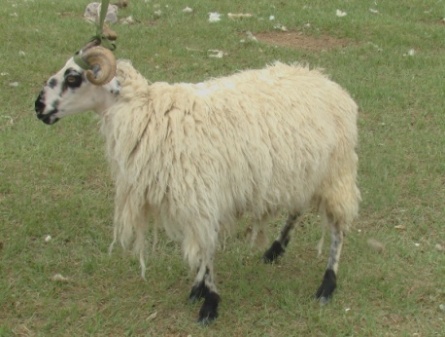 |
| TIBP | 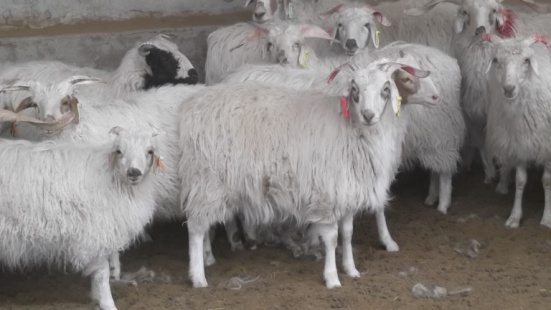 |
