# Supplementary material for: Genome-wide analysis reveals population structure and selection in Chinese indigenous sheep breeds
Source: BMC Genomics. 2015 Mar 17;16(1):194. doi: 10.1186/s12864-015-1384-9 (PMC4404018; doi:10.1186/s12864-015-1384-9)
Supplement: Additional file 11: Table S6. — Annotation of consecutive selection regions on OAR2 and OAR6 of Duolang sheep. [file 12864_2015_1384_MOESM11_ESM.docx]

**Table S6 Annotation of consecutive selection regions on OAR2 and OAR 6 of Duolang sheep**

| **chr** | **breed** | **Di** | **window.no** | **start** | **end** | **region** |  | **annotation gene** |
| --- | --- | --- | --- | --- | --- | --- | --- | --- |
| 2 | DUL | 10.90156 | 1081 | 51300000 | 51600000 | 51189786 | 51317574 | ZCCHC7 |
| 2 | DUL | 10.90156 | 1081 | 51300000 | 51600000 | 51353496 | 51543382 | PAX5 |
| 2 | DUL | 14.76874 | 1082 | 51600000 | 51900000 | 51689236 | 51755300 | MELK |
| 2 | DUL | 14.76874 | 1082 | 51600000 | 51900000 | 51855253 | 52042051 | GNE |
| 2 | DUL | 19.02471 | 1083 | 51900000 | 52200000 | 52048202 | 52065307 | CLTA |
| 2 | DUL | 19.02471 | 1083 | 51900000 | 52200000 | 52087650 | 52089416 | CCIN |
| 2 | DUL | 19.02471 | 1083 | 51900000 | 52200000 | 52094611 | 52116621 | GLIPR2 |
| 2 | DUL | 19.02471 | 1083 | 51900000 | 52200000 | 52128947 | 52224617 | RECK |
| 2 | DUL | 32.22626 | 1084 | 52200000 | 52500000 | 52379896 | 52380737 | OR13C4 |
| 2 | DUL | 32.22626 | 1084 | 52200000 | 52500000 | 52384173 | 52403379 | TMEM8B |
| 2 | DUL | 32.22626 | 1084 | 52200000 | 52500000 | 52411021 | 52417200 | C9ORF128 |
| 2 | DUL | 32.22626 | 1084 | 52200000 | 52500000 | 52421111 | 52423389 | HINT2 |
| 2 | DUL | 32.22626 | 1084 | 52200000 | 52500000 | 52424272 | 52426475 | SPAG8 |
| 2 | DUL | 32.22626 | 1084 | 52200000 | 52500000 | 52426982 | 52460167 | NPR2 |
| 2 | DUL | 32.22626 | 1084 | 52200000 | 52500000 | 52480200 | 52481163 | MSMP |
| 2 | DUL | 32.22626 | 1084 | 52200000 | 52500000 | 52481502 | 52484489 | RGP1 |
| 2 | DUL | 32.22626 | 1084 | 52200000 | 52500000 | 52485576 | 52495944 | GBA2 |
| 2 | DUL | 27.68468 | 1085 | 52500000 | 52800000 | 52496387 | 52500153 | CREB3 |
| 2 | DUL | 27.68468 | 1085 | 52500000 | 52800000 | 52506555 | 52516651 | TLN1 |
| 2 | DUL | 27.68468 | 1085 | 52500000 | 52800000 | 52537491 | 52544000 | TPM2 |
| 2 | DUL | 27.68468 | 1085 | 52500000 | 52800000 | 52546246 | 52551800 | CA9 |
| 2 | DUL | 27.68468 | 1085 | 52500000 | 52800000 | 52564548 | 52567008 | CCDC107 |
| 2 | DUL | 27.68468 | 1085 | 52500000 | 52800000 | 52570300 | 52570623 | PAGE4 |
| 2 | DUL | 27.68468 | 1085 | 52500000 | 52800000 | 52572817 | 52573775 | SIT1 |
| 2 | DUL | 27.68468 | 1085 | 52500000 | 52800000 | 52594675 | 52601292 | CD72 |
| 2 | DUL | 27.68468 | 1085 | 52500000 | 52800000 | 52603694 | 52609309 | TESK1 |
| 2 | DUL | 27.68468 | 1085 | 52500000 | 52800000 | 52614999 | 52620411 | FAM166B |
| 2 | DUL | 27.68468 | 1085 | 52500000 | 52800000 | 52619334 | 52656644 | RUSC2 |
| 2 | DUL | 27.68468 | 1085 | 52500000 | 52800000 | 52695717 | 52777483 | ATP8B5 |
| 2 | DUL | 18.17292 | 1086 | 52800000 | 53100000 | 52819198 | 52987887 | UNC13B |
| 2 | DUL | 18.17292 | 1086 | 52800000 | 53100000 | 52875396 | 52875743 | RPS26 |
| 2 | DUL | 18.17292 | 1086 | 52800000 | 53100000 | 53036658 | 53059144 | KIAA1539 |
| 2 | DUL | 18.17292 | 1086 | 52800000 | 53100000 | 53061137 | 53064352 | STOML2 |
| 2 | DUL | 18.17292 | 1086 | 52800000 | 53100000 | 53070412 | 53076980 | PIGO |
| 2 | DUL | 18.17292 | 1086 | 52800000 | 53100000 | 53079030 | 53084369 | FANCG |
| 2 | DUL | 18.17292 | 1086 | 52800000 | 53100000 | 53089788 | 53099744 | VCP |
| 2 | DUL | 31.4677 | 1087 | 53100000 | 53400000 | 53140327 | 53150606 | DNAJB5 |
| 2 | DUL | 31.4677 | 1087 | 53100000 | 53400000 | 53159612 | 53165457 | KIAA1045 |
| 2 | DUL | 31.4677 | 1087 | 53100000 | 53400000 | 53231881 | 53238068 | C9ORF144B |
| 2 | DUL | 21.05771 | 1091 | 54300000 | 54600000 | 54512991 | 54518180 | FAM75D1 |
| 2 | DUL | 23.73333 | 1092 | 54600000 | 54900000 | 54714979 | 54804797 | TLE1 |
| 2 | DUL | 29.77657 | 1094 | 55200000 | 55500000 | 55213240 | 55213416 | MT-CO1 |
| 2 | DUL | 29.77657 | 1094 | 55200000 | 55500000 | 55215628 | 55215873 | MT-ND1 |
| 2 | DUL | 26.94011 | 1095 | 55500000 | 55800000 | 55565702 | 55566184 | RPL21 |
| 2 | DUL | 17.57322 | 1098 | 56400000 | 56700000 | 56637335 | 56817586 | TLE4 |
| 2 | DUL | 11.71046 | 1104 | 58200000 | 58500000 | 58283842 | 58285156 | PSMD14 |
| 2 | DUL | 11.71046 | 1104 | 58200000 | 58500000 | 58446152 | 58656152 | GNAQ |
| 2 | DUL | 16.38318 | 1105 | 58500000 | 58800000 | 58679887 | 58874987 | GNA14 |
| 2 | DUL | 11.41543 | 1107 | 59100000 | 59400000 | 58890630 | 59185647 | VPS13A |
| 2 | DUL | 11.41543 | 1107 | 59100000 | 59400000 | 59266687 | 59654654 | PRUNE2 |
| 6 | DUL | 15.41522 | 3326 | 30000000 | 30300000 | 29907812 | 30013548 | PDLIM5 |
| 6 | DUL | 15.41522 | 3326 | 30000000 | 30300000 | 30282223 | 30307578 | HPGDS |
| 6 | DUL | 20.97406 | 3327 | 30300000 | 30600000 | 30314536 | 30402687 | SMARCAD1 |
| 6 | DUL | 11.89725 | 3328 | 30600000 | 30900000 | 30599335 | 30659954 | MAK16 |
| 6 | DUL | 11.89725 | 3328 | 30600000 | 30900000 | 30679519 | 30679993 | TMEM186 |
| 6 | DUL | 11.89725 | 3328 | 30600000 | 30900000 | 30708618 | 30709169 | ATOH1 |
| 6 | DUL | 11.89725 | 3328 | 30600000 | 30900000 | 30768380 | 30816210 | GRID2 |
| 6 | DUL | 17.85559 | 3333 | 32100000 | 32400000 | 32284857 | 32288475 | CFDP2 |
| 6 | DUL | 10.76918 | 3338 | 33600000 | 33900000 | 33776934 | 33887406 | FAM190A |
| 6 | DUL | 25.06858 | 3347 | 36600000 | 36900000 | 36566367 | 36625174 | PKD2 |
| 6 | DUL | 25.06858 | 3347 | 36600000 | 36900000 | 36651734 | 36657086 | SPP1 |
| 6 | DUL | 25.06858 | 3347 | 36600000 | 36900000 | 36804948 | 36817206 | MEPE |
| 6 | DUL | 25.06858 | 3347 | 36600000 | 36900000 | 36837847 | 36866809 | IBSP |
| 6 | DUL | 13.61187 | 3349 | 37200000 | 37500000 | 37194092 | 37257047 | FAM184B |
| 6 | DUL | 13.61187 | 3349 | 37200000 | 37500000 | 37278639 | 37505383 | LCORL |
| 6 | DUL | 13.61187 | 3349 | 37200000 | 37500000 | 37289107 | 37333851 | NCAPG |
| 6 | DUL | 11.95574 | 3351 | 37800000 | 38100000 | 38079006 | 38139267 | SET |
| 6 | DUL | 11.12369 | 3357 | 39600000 | 39900000 | 39692231 | 39707830 | SLIT2 |
